# Genotyping-by-Sequencing Derived High-Density Linkage Map and its Application to QTL Mapping of Flag Leaf Traits in Bread Wheat

**DOI:** 10.1038/s41598-017-16006-z

**Published:** 2017-11-27

**Authors:** Waseem Hussain, P. Stephen. Baenziger, Vikas Belamkar, Mary J. Guttieri, Jorge P. Venegas, Amanda Easterly, Ahmed Sallam, Jesse Poland

**Affiliations:** 1 Department of Agronomy and Horticulture, University of Nebraska, Lincoln, NE 68583 USA; 2 USDA, Agricultural Research Service, Center for Grain and Animal Health Research, Hard Winter Wheat Genetics Research Unit, 1515 College Avenue, Manhattan, KS 66502 USA; 3 Department of Genetics, Faculty of Agriculture, Assiut University, 71526 Assiut, Egypt; 4 Wheat Genetics Resource Center, Department of Plant Pathology, Kansas State University, Manhattan, KS 66506 USA

## Abstract

Winter wheat parents ‘Harry’ (drought tolerant) and ‘Wesley’ (drought susceptible) were used to develop a recombinant inbred population with future goals of identifying genomic regions associated with drought tolerance. To precisely map genomic regions, high-density linkage maps are a prerequisite. In this study genotyping-by- sequencing (GBS) was used to construct the high-density linkage map. The map contained 3,641 markers distributed on 21 chromosomes and spanned 1,959 cM with an average distance of 1.8 cM between markers. The constructed linkage map revealed strong collinearity in marker order across 21 chromosomes with POPSEQ-v2.0, which was based on a high-density linkage map. The reliability of the linkage map for QTL mapping was demonstrated by co-localizing the genes to previously mapped genomic regions for two highly heritable traits, chaff color, and leaf cuticular wax. Applicability of linkage map for QTL mapping of three quantitative traits, flag leaf length, width, and area, identified 21 QTLs in four environments, and QTL expression varied across the environments. Two major stable QTLs, one each for flag leaf length (*Qfll*.*hww-7A*) and flag leaf width (*Qflw*.*hww-5A*) were identified. The map constructed will facilitate QTL and fine mapping of quantitative traits, map-based cloning, comparative mapping, and in marker-assisted wheat breeding endeavors.

## Introduction

Bread wheat (*Triticum aestivum* L.) is the staple food crop for more than 35% of the global human population and accounts for 20% of all calories consumed by humans throughout the world^1^. Identifying genes for most agronomic traits in wheat is challenging due to the complexity of the traits arising due to polygenic nature of genes, their interaction with the environment and polyploidy nature of wheat^2,3^.

Quantitative trait loci (QTL) mapping using bi-parental populations is one of the key approaches to dissect the complex traits and identify genomic regions underlying quantitative traits for breeding purposes^4^. The success of QTL mapping largely depends upon the population size, type of markers, and density of markers used for genetic mapping^5^. In wheat, enormous efforts have been invested in the last two decades to dissect the complex traits using QTL mapping approach^6^. However, most of the bi-parental QTL mapping studies used low-density markers, thus do not provide the accurate information about the number and location of the QTLs controlling the traits. For better precision of QTL mapping, high-density linkage maps based on high-throughput markers like SNPs (single nucleotide polymorphism) is necessary. High-density linkage maps can increase the precision of effect estimates of QTL detected, provides accurate position of QTL and may lead to the identification of candidate genes underlying the complex traits^7^. Recently, next generation sequencing (NGS) technologies have paved the way for the development of sequence-based SNP markers that combines advantages of time and cost effectiveness, dense marker coverage at a genome-wide level, high mapping resolution, and more comparable genome and genetic maps among mapping populations^8^. Among the various next generation sequencing technologies, genotyping-by-sequencing (GBS) is one of the NGS-based method used for simultaneous SNP discovery and genotyping^9,10^. The power of the GBS in the development of high-density linkage maps and QTL mapping in various crops including wheat^11–13^ has been recently highlighted.

In the construction of high-density linkage map based on high-throughput SNP markers, genotyping errors including heterozygosity, excessive single cross events, unexpected double recombinants, segregation distortion and allele switching are common^14^. These genotyping errors can distort the linkage maps, especially by expanding the map distance due to overestimation of recombination frequencies^15^. Moreover, genotyping errors are naturally associated with the GBS data^16^. It is always prudent to check for these genotyping errors to ensure that best quality genotypes and markers are being used for linkage map construction. Previously, in the construction of high-density linkage maps in various crop researchers have mainly focused on missing data percentage and heterozygosity as the main contributors to genotyping errors and largely ignore the errors arising due to excessive single cross events or double recombinants. In this study, we used a robust procedure to check and visualize the genotyping errors comprehensively and ensure high-quality genotypes and markers were retained for final linkage map construction.

In wheat, the flag leaf is the primary source of carbohydrates stored in grains, and the flag leaf plays a significant role in facilitating photosynthesis and determining yield potential^17^. For increasing grain yield, it is necessary to understand the genetic mechanism underlying flag leaf-related traits. Flag leaf-related traits are complex in nature controlled by many genes and strongly influenced by environmental factors^18^. In wheat, numerous studies on QTL mapping for flag leaf-related traits have been reported^18–20^. However, only one study has investigated flag leaf-related traits using high-density linkage map based on high-throughput SNP markers^21^.

In this study, we used a RIL mapping population derived from two contrasting parents, ‘Harry’ and ‘Wesley’ for high-density linkage map construction. Harry is white chaffed hard red winter wheat adapted to rainfed conditions and is drought tolerant cultivar^22^. Wesley is bronze chaffed hard red winter wheat adapted to irrigated conditions and is drought susceptible^23^. The population is unique and was developed with a the long term goal to identify QTLs for various agronomic traits associated with drought adaptive features and grain yield in Harry and Wesley and to breed these traits into desirable cultivars in Great Plains of U.S. To precisely map the genomic regions in this RIL population for various agronomic traits, a high-density linkage map will be prerequiste.

The present study was undertaken to (i) construct high-density linkage map based on SNPs generated through GBS in recombinant inbred lines (RILs) of bread wheat derived from cross between highly contrasting parents Harry (drought tolerant hard red winter wheat cultivar) and Wesley (drought susceptible hard red winter wheat cultivar), (ii) investigate the marker order of the constructed linkage map with the POPSEQ (Population Sequencing)- based high-density linkage map, (iii) determine the accuracy and reliability of the constructed linkage map by mapping known genomic regions of two highly heritable traits of chaff color (CC) and leaf cuticular wax (CW), and (iv) demonstrate the applicability of linkage map to identify QTL and QTL x environment interactions for three quantitative traits, flag leaf length (FLL), flag leaf width (FLW), and flag leaf area (FLA). The high-density linkage map constructed contained 3,641 markers distributed on 21 chromosomes. Before the construction of final linkage map, we used robust filtering parameters based on efficient and effective algorithms in R/qtl^24^ and R/ASMap^25^ packages in R-software^26^ to check and remove the low-quality genotypes and markers. Various diagnostic tools in a greater depth were used to assess the quality of linkage. The accuracy and reliability of linkage map for QTL mapping were confirmed by co-locating the QTLs for CC and CW at their expected locations in the wheat genome. The high-density linkage map developed in this study may be a useful asset to dissect the quantitative traits and fine map the key genomic regions related to drought and adaptation in Harry x Wesley derived RIL population. It may also provide the basis for the next phase of research such as QTL mapping (as demonstrated for flag leaf traits in this study), comparative mapping, and in marker-assisted breeding endeavors.

## Results

### Linkage map construction

In the first stage of mapping (before error correction/filtering for low-quality markers), linkage groups (LGs) formed ranged in size from 43 cM to 846 cM with a total map length of 7,269 cM. The total map length obtained was vastly overinflated as compared to previously derived GBS linkage maps in bread wheat^9,27,28^. In the development of high-density linkage maps, over-inflation of linkage map lengths is a well-known phenomenon due to the effect of genotyping errors (unexpected double recombinants). Besides genotyping errors, high percentages of missing data and distorted markers may also affect the mapping distance. To address these issues, markers were inspected, and potential genotyping errors or inflated number of double recombinants and distorted markers were removed (supplementary methods). Graphical representation of genotypes, pairwise recombination fraction between all pairs of markers and heat map obtained before error correction and after error correction (Supplementary Figs S2,S3, and S4) were used as diagnostic tools to evaluate and verify the quality of linkage map. Graphical representation of genotypes reflects the number of possible double crossovers, which convey potential genotyping errors. Recombination blocks were clearly defined in the filtered data set as compared to the unfiltered dataset (Supplementary Fig. S2), indicating that the genotypic dataset used to construct final linkage map was well-corrected and suitable for linkage mapping. In the pairwise recombination fraction plot (Supplementary Fig. S3) higher recombination fraction values and higher LOD score was found for some of the markers that are unrealistic indicating a potential problem with these markers and were discarded during the filtering process. Also, a heat map of pairwise recombination and LOD score between all pairs of markers was visualized to check the quality of constructed linkage map. The heat map that combines the estimates of pairwise recombination fraction between markers as well as LOD scores reflects the strength of linkage between markers and can be used to find marker ordering errors. The strong recombination fraction and LOD score along the diagonal and lack of it in off-diagonal in error corrected data set (Supplementary Fig. S4 (b)) indicated the robustness of the developed linkage map.

**Figure 1:**
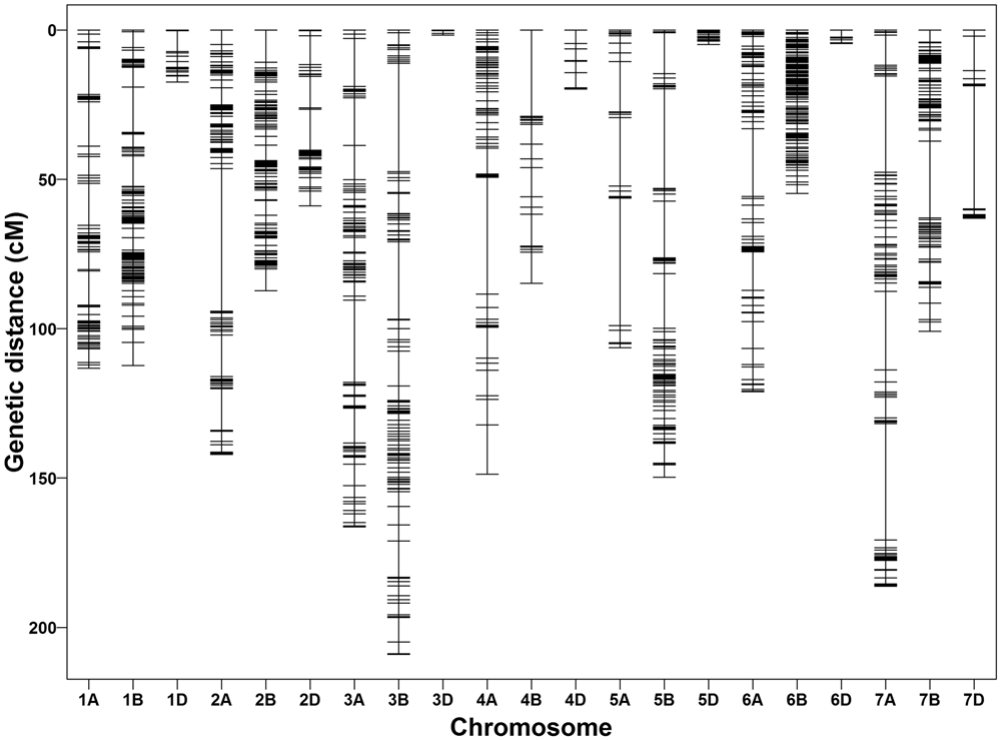
Linkage map constructed from genotyping-by-sequencing derived SNPs in a recombinant inbred population derived from a cross between Harry and Wesley.

**Figure 2:**
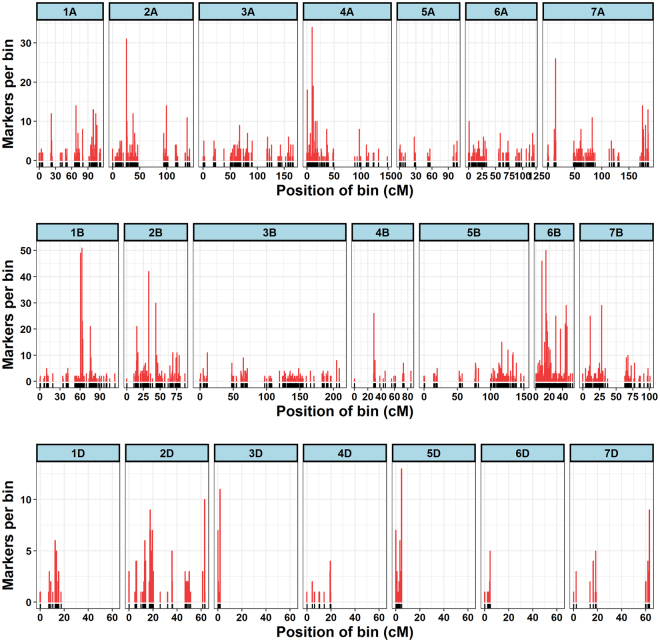
Distribution of marker bins on 21 chromosomes of the linkage map. The x-axis shows the position of bin on each chromosome and y-axis shows the number of markers in each bin.

In the second stage of mapping, final linkage map was constructed having 3,641 high-quality SNP markers distributed on 21 chromosomes (Fig. 1). These 3,641 SNPs grouped into 684 unique bins (Table 1 and Supplementary Data S1). Marker name, position within reference scaffold, marker alleles, reference chromosome, genetic position (cM), and color-coded genotypes for each marker are provided in Supplementary Data S1. The linkage map spanned a total length of 1,959.3 cM with an average marker interval of 2.8 cM and average marker density of 1.8 cM per marker (Table 1). The number of genetic bins on each chromosome varied from 2 (6D) to 76 (6B) (Fig. 2) and markers from 11 (6D) to 577 (6B) per chromosome. The shortest chromosome was 3D, which harbored 21 markers with a genetic length of 1.7 cM, an average marker interval of 0.57 cM and marker density of 12.2 cM per marker. The longest chromosome was 3B, and it harbored 223 markers with a genetic length of 209.1 cM, an average marker interval of 3.6 cM and marker density of 1.07 cM per marker. Marker density was lowest in 3D (12.2 cM per marker) followed by 6B (10.5 cM per marker) and highest in 5A (0.47 cM per marker) and 7D (0.63 cM per marker). In this linkage map, we noticed five gaps greater than 30 cM on 2A, 5A, 5B, 6A, and 7A, respectively. The A, B and D genomes harbored 1325, 2033 and 283 markers with a total length of 984.1 cM, 798.6 cM, and 176.5 cM. Among the three genomes of wheat, the B genome has lowest marker density (2.5 cM per marker) and the most markers (55.8%), followed by the D genome (1.6 cM per marker and 7.7% of total markers) and A genome (1.3 cM per marker and 36.3% of total markers) genome (Table 1).

**Table 1:** Marker statistics of the linkage map constructed from recombinant inbred lines derived from cross between Harry and Wesley.

Chromosome	No. of markers	% markers	Bins	Total length (cM)	Average marker/bin interval	Marker density	SDM^1^	% SDM^2^	SDR^3^
1A	209	5.74	40	113.18	2.83	1.85	43	15.09	4
1B	338	9.28	52	112.29	2.16	3.01	292	42.14	15
1D	30	0.82	7	17.38	2.48	1.73	5	11.90	0
2A	231	6.34	41	142.12	3.47	1.63	49	15.17	4
2B	352	9.67	57	87.22	1.53	4.04	68	13.49	4
2D	122	3.30	26	65.30	2.51	1.87	14	8.92	1
3A	182	5.00	40	166.34	4.16	1.09	31	12.11	0
3B	223	6.12	58	209.03	3.60	1.07	64	16.49	4
3D	21	0.58	3	1.72	0.57	12.21	64	71.91	2
4A	233	6.40	37	148.75	4.02	1.57	75	19.89	4
4B	73	2.00	12	84.75	7.06	0.86	33	27.97	2
4D	15	0.41	3	19.82	6.61	0.76	15	36.59	2
5A	50	1.37	14	106.41	7.60	0.47	27	21.09	2
5B	223	6.12	53	149.76	2.83	1.49	94	22.17	8
5D	44	1.21	8	4.8	0.60	9.17	11	16.92	1
6A	162	4.45	45	121.12	2.69	1.34	51	18.21	2
6B	577	15.85	76	54.73	0.72	10.54	112	13.48	4
6D	11	0.30	2	4.49	2.25	2.45	19	35.85	1
7A	258	7.09	51	186.22	3.65	1.39	164	30.04	8
7B	247	6.78	50	100.87	2.02	2.45	45	10.82	4
7D	40	1.10	9	63.07	7.01	0.63	27	25.96	2
A genome	1325	36.39	268	984.14	3.68	1.35	440	33.78	24
B genome	2033	55.84	358	798.65	2.23	2.55	708	54.33	41
D genome	283	7.78	58	176.58	3.05	1.60	155	11.89	10
Total	3641		684	1959.37	2.86	1.86	1303	21.29	75

^1^Number of segregation distorted markers. ^2^Percentage of segregation distorted markers. ^3^Number of segregation distorted regions.

### Segregation distortion

**Figure 3:**
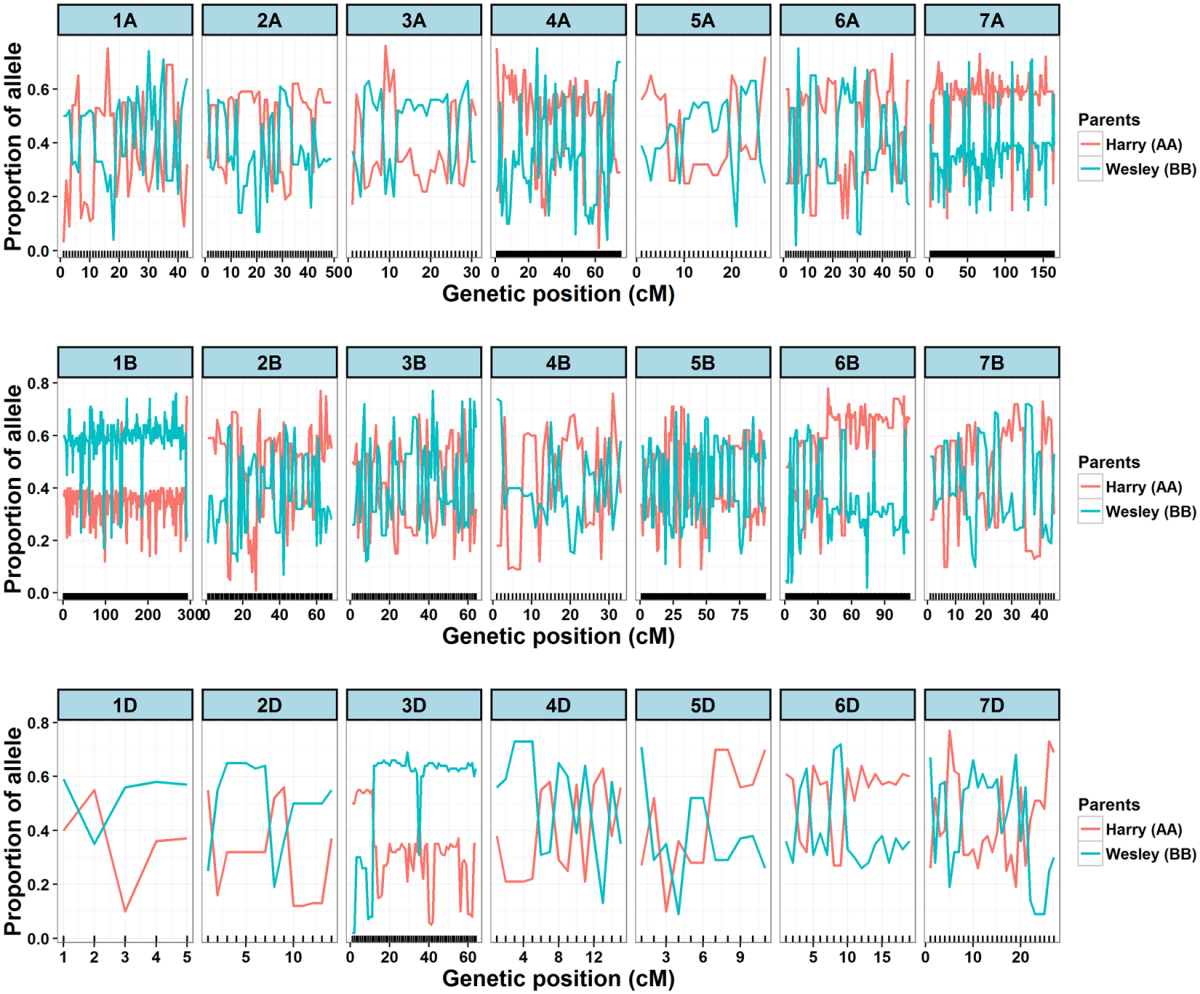
The proportion of contributing alleles Harry (AA) and Wesley (BB) for each marker. Parental allele contributed by Harry and Wesley is represented in red and blue. Chromosome name is given on top and x-axis represents the position of distorted markers.

**Figure 4:**
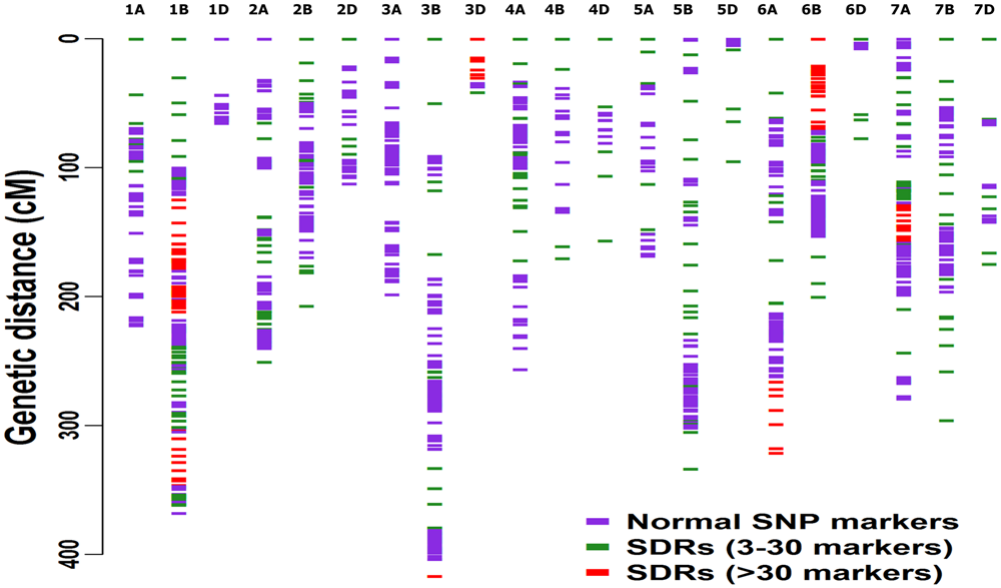
Distribution of genotyping-by-sequencing derived normal (blue) and segregation distorted regions (SDRs) on 21 chromosomes of the linkage map. For better visualization, SDRs are colored as green (3–30 markers clustered together) and red (>30 markers clustered together) depending upon the number of markers clustered together to form SDRs.

SNP calling followed by filtering (see methods section for details) resulted in 6,120 markers for the construction of linkage map. Of the 6,120 markers, 1,303 (21.2%) showed significant (p < 0.01) distortion (Table 1) with marker alleles biased towards either parent (Fig. 3). Segregation distortion markers varied considerably, and chromosome 3D (64 segregation distortion markers) and 1B (292) revealed the most severe segregation distortion, with 71.9% and 42.1% of markers distorted, respectively. Chromosomes 2D (14) and 7B (45) showed the lowest segregation distortion, with 8.9% and 10.8% of markers distorted, respectively. Among the 1,303 segregation distortion markers, 708 (54.3%) of them were present in the B genome, 440 (33.7%) in the A genome and 155 (11.8%) in the D genome (Table 1). In the linkage map, segregation distortion markers tend to be clustered (Fig. 4), rather than randomly distributed across a chromosome and clusters of three or more consecutive adjacent distorted markers were defined as ‘segregation distorted regions.’ In total, we found 75 segregation distorted regions, with 24 of segregation distorted regions present in the A genome, 41 in B and 10 in D. Segregation distorted regions in the map varied from 15 (1B) to 0 (1D and 3 A). Most of the segregation distorted regions on chromosomes 1B, 3D, 6 A, 6B, and 7 A occurred in large clusters (>30 markers) (Fig. 4), with marker alleles mainly biased towards male (Wesley) parent (Fig. 3).

### Comparison of linkage map with POPSEQ-v2.0 based on the high-density linkage map

**Figure 5:**
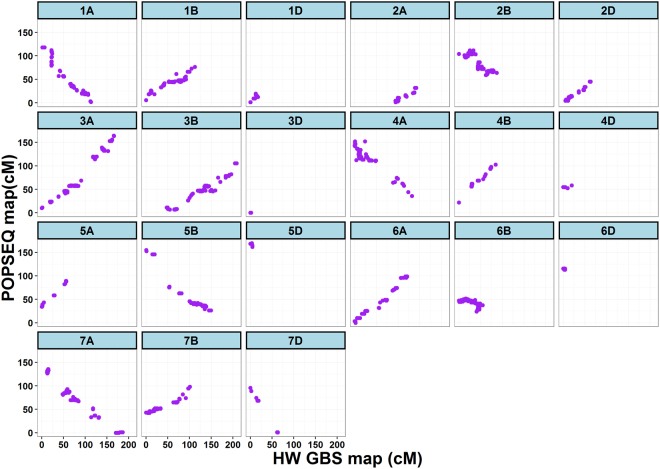
Dot plot depicting the marker order collinearity between Harry x Wesley map (cM) and POPSEQ-based on high-density linkage map (cM) for 21 chromosomes of hexaploid wheat.

We placed 2,581 SNPs (70.8%) out of 3,641 SNPs in International Wheat Genome Sequencing Consortium (IWGSC) contigs that were anchored to high-density linkage map developed by Chapman *et al*.^29^. We found a strong collinear relationship in marker order on the 21 chromosomes between our constructed linkage map and POPSEQ v2.0 (Fig. 5) confirming the high quality of linkage map built in this study.

### The reliability of the linkage map for QTL mapping

**Figure 6:**
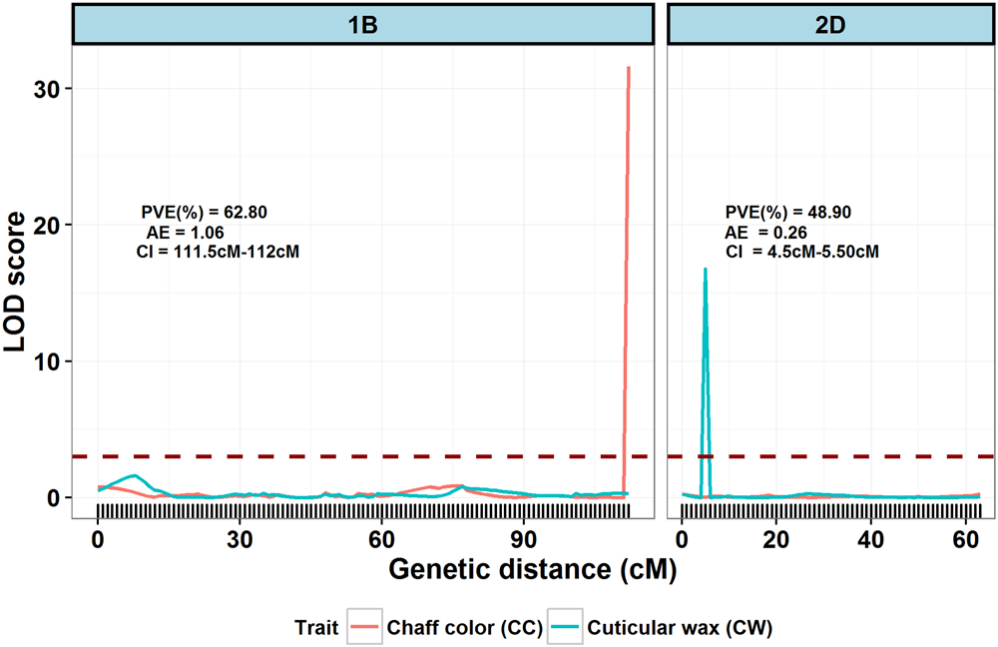
Graphical display of significant QTL for chaff color (CC) and leaf cuticular wax (CW) on chromosome 1B and 2D. Phenotypic variance explained (PVE), additive effect (AE) and confidence interval (CI) for identified QTL is shown on top of the figure for both traits. The dashed dark-red horizontal line indicates the threshold LOD score of 3 to declare significant QTL.

Once a high-density linkage map is developed using high-throughput SNP markers, we are not sure how accurate is the linkage map regarding marker position and location on chromosomes, as well as for QTL mapping purposes. This linkage map accuracy is significant in the case of bread wheat due to non-availability of reliable reference genome. To evaluate the accuracy and reliability of a linkage map for QTL mapping, QTL analysis was performed for two highly heritable traits including CC and CW. The main purpose to study CC and CW was to evaluate the quality of the high-density linkage map. A major QTL (named *Qcc*.*hww-1B*) for CC was identified on chromosome 1B (Fig. 6). This QTL was located in the confidence interval between marker *GBSHW6*2*2* and *GBSHW646*, with LOD score of 31.6, and explained 62.8% of the phenotypic variance (PVE) in the RIL population, with the bronze chaff color allele coming from Wesley parent. The QTL mapping for CW located a single major QTL (*Qcw*.*hww-2D*) in the confidence interval between marker *GBSHW1918* and *GBSHW1871*, with LOD score of 16.77, and explained 48.9% of the phenotypic variance in the RIL population, with the wax contributing allele coming from Harry parent (Fig. 6). Mapping QTL of CC and CW exactly on the same chromosome and genomic regions indicates linkage map constructed of high quality and reliable for QTL mapping experiments.

### Phenotypic evaluation

The Harry x Wesley derived RIL population including parents showed variation for FLL, FLW, and FLA. The descriptive statistics and frequency distribution averaged over all the four environments (2 years and 2 locations) for FLL, FLW and FLA is shown in Supplementary Table S1 and Supplementary Fig. S5. Harry revealed higher mean values than the other parent Wesley for all the traits across all environments. The average mean values of lines varied from 16.7 cm to 28.5 cm for FLL, 1.2 cm to 2.0 cm for FLW and 18.3 cm to 43.3 cm for FLA (Supplementary Table S1). The coefficient of variation (CV) was higher for FLA (17%) and lower for FLW (10%). Transgressive segregation was observed for all observed traits across all environments, with trait values of RILs being larger or smaller than that of parents, suggesting that alleles with positive effects are distributed among both the parents. Analysis of variance (ANOVA) showed significant differences (P < 0.01) among lines for all the traits, indicating high levels of variation in the RIL population (Supplementary Table S2). The environment (location x line and year x line) interactions (P < 0.05) was significant for FLL and FLA. Entry-mean heritability of 74%, 78%, and 72% were observed for FLL, FLW, and FLA, respectively.

### QTL analysis for FLL, FLW and FLA traits

**Figure 7:**
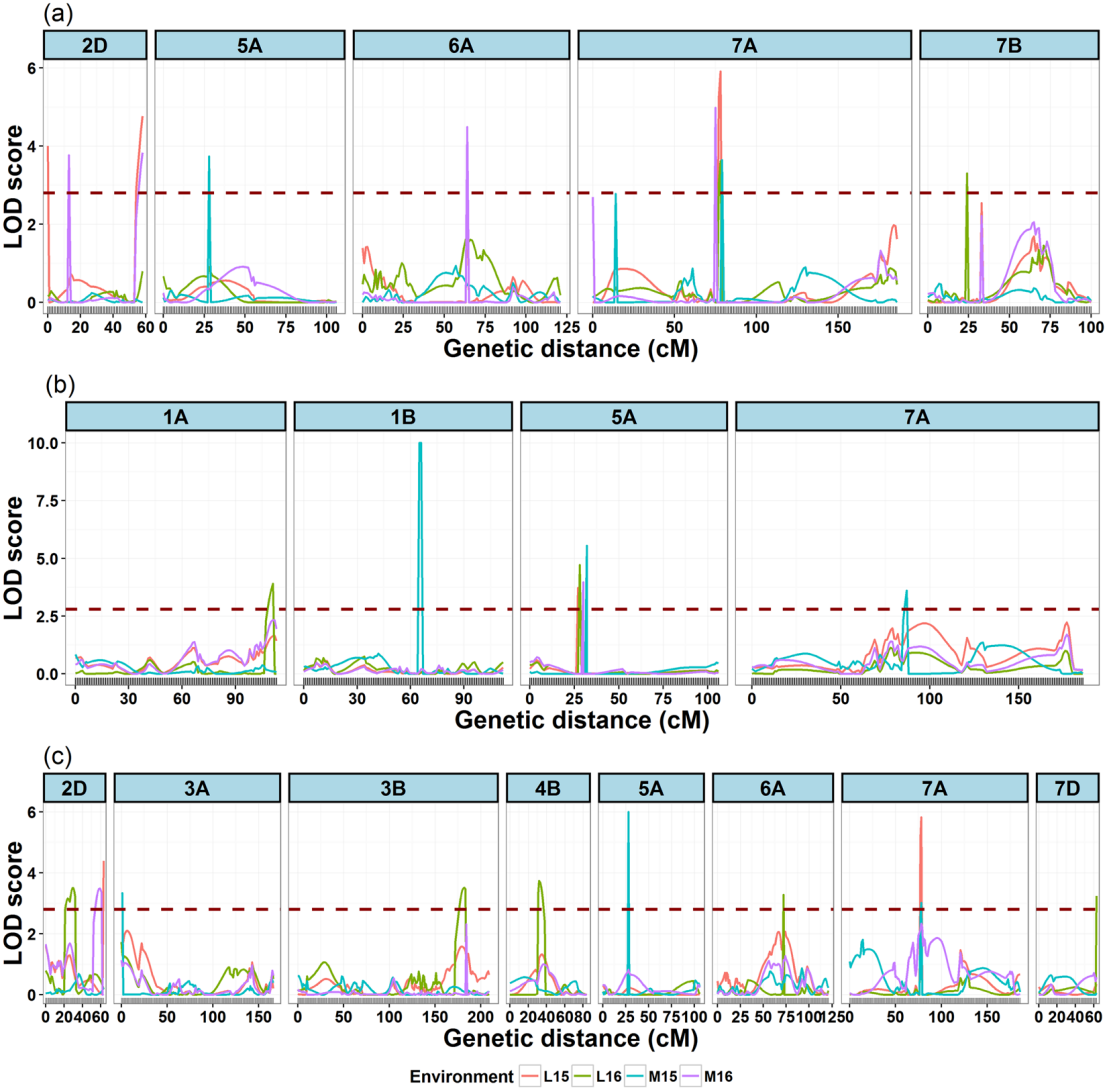
Graphical display of significant QTL detected in 4 environments for (**a**) flag leaf length (FLL), (**b**) flag leaf width (FLW) and (**c**) flag leaf area (FLA). Red, green, blue and violet represent the four environments (L15, L16, M15, and M16). The dashed dark-red horizontal line indicates the threshold LOD score of 3 to declare significant QTL.

A total of 21 additive QTL associated with FLL, FLW, and FLA were detected in the four environments- Lincoln, Nebraska in 2015-2016 (L15 and L16) and Mead, Nebraska in 2015-2016 (M15 and M16) (Table 2 and Fig. 7). These QTL were distributed across ten chromosomes (1A, 1B, 2D, 3B, 4B, 5A, 6A, 7A, 7B, and 7D) and individually explained 5.2 to 16.5% of the phenotypic variance. Among the 21 QTL, 13 (61%) loci involved alleles from Harry for increasing phenotypic values, whereas the other 8 (39%) loci had alleles from Wesley for decreasing phenotypic values, indicated that positive alleles for FLL, FLW and FLA were present in both the parents.

**Table 2:** List of significant QTL detected in four environments for flag leaf length (FLL), flag leaf width (FLW) and flag leaf area (FLA) using high-density linkage map.

Trait	Env.^1^	QTL	Chr.^2^	Pos^3^	Flanking markers	LOD	PVE^4^	Add^5^
FLL	L15	*Qfll*.*hww-2D*.*a*	2D	0	*HWGBS1994-HWGBS1859*	4.00	9.65	3.51
		*Qfll*.*hww-2D*.*b*	2D	58	*HWGBS1918-HWGBS1863*	4.77	9.59	3.56
		*Qfll*.*hww*.*6A*	6A	64	*HWGBS3922-HWGBS3920*	3.77	9.32	3.49
		*Qfll*.*hww*.*7A*	7A	78	*HWGBS5487-HWGBS5600*	5.91	14.22	3.64
	L16	*Qfll*.*hww-2D*.*c*	2D	13	*HWGBS1948-HWGBS1887*	3.74	8.91	3.52
		*Qfll*.*hww-7A*	7A	78	*HWGBS5487-HWGBS5600*	3.61	9.50	3.51
		*Qfll*.*hww-7B*	7B	24	*HWGBS5671-HWGBS5669*	3.30	8.72	3.48
	M15	*Qfll*.*hww-5A*	5A	28	*HWGBS3329-HWGBS3337*	3.74	7.93	−3.34
		*Qfll*.*hww-7A*	7A	78	*HWGBS5487-HWGBS5600*	3.64	7.83	3.34
	M16	*Qfll*.*hww-2D*.*c*	2D	13	*HWGBS1948-HWGBS1887*	3.76	8.46	3.3
		*Qfll*.*hww-2D*.*b*	2D	58	*HWGBS1918-HWGBS1863*	3.84	8.66	3.31
		*Qfll*.*hww-6A*	6A	64	*HWGBS3922-HWGBS3920*	4.49	9.55	3.33
		*Qfll*.*hww-7A*.*b*	7A	75	*HWGBS5458-HWGBS5471*	4.98	10.62	3.35
FLW	L15	*Qflw*.*hww-5A*	5A	28	*HWGBS3329-HWGBS3337*	3.72	10.28	−1.01
	L16	*Qflw*.*hww-1A*	1A	111	*HWGBS136-HWGBS115*	3.90	9.97	−1.03
		*Qflw*.*hww-5A*	5A	28	*HWGBS3329-HWGBS3337*	4.71	10.94	−1.03
	M15	*Qflw*.*hww-1B*	1B	66	*HWGBS799-HWGBS805*	14.54	16.53	1.04
		*Qflw*.*hww-5A*	5A	28	*HWGBS3329-HWGBS3337*	5.55	6.95	−1.02
		*Qflw*.*hww-7A*	7A	87	*HWGBS5576-HWGBS5480*	3.61	5.21	1.02
	M16	*Qflw*.*hww-5A*	5A	28	*HWGBS3329-HWGBS3337*	3.96	10.86	−1.04
FLA	L15	*Qfla*.*hww-2D*.*a*	2D	63	*HWGBS1860-HWGBS1994*	4.39	11.02	3.73
		*Qfla*.*hww-7A*	7A	78	*HWGBS5487-HWGBS5600*	5.83	14.57	3.86
	L16	*Qfla*.*hww-2D*.*b*	2D	30	*HWGBS1929-HWGBS1851*	3.50	8.28	3.95
		*Qfla*.*hww-3B*	3B	182	*HWGBS2422-HWGBS2280*	3.51	8.32	−3.96
		*Qfla*.*hww-4B*	4B	32	*HWGBS3140-HWGBS3186*	3.74	8.23	3.95
		*Qfla*.*hww-6A*	6A	72	*HWGBS3975-HWGBS3938*	3.28	7.59	3.9
		*Qfla*.*hww-7D*	7D	63	*HWGBS6027-HWGBS6033*	3.24	7.20	−3.87
	M15	*Qfla*.*hww-3A*	3A	0	*HWGBS2032-HWGBS2072*	3.35	7.09	−3.78
		*Qfla*.*hww-5A*	5A	28	*HWGBS3329-HWGBS3337*	7.98	15.02	−4.24
	M16	*Qfla*.*hww-2D*.*c*	2D	59	*HWGBS1931-HWGBS1938*	3.49	9.60	3.78

^1^Environment. ^2^Chromosome where the QTL was located. ^3^Chromosome position (cM) of the QTL. ^4^Phenotypic variance explained. ^5^Additive effect (positive values of the additive effect indicated that alleles from parent ‘Harry’ were in the direction of increasing trait score and negative indicated that alleles from parent ‘Wesley’ were in the direction of increasing trait score).

For FLL, a total of 7 additive effect QTL were detected explaining from 7.8% to 14.2% of phenotypic variance. These QTLs were distributed over five chromosomes, which were 2D, 5A, 6A, 7A and 7B. Most of the QTLs identified had small effects (7.8 to 9.6%). Four QTL were identified in two or more than two environments. The QTL *Qfll*.*hww-7A* flanked by markers *HWGBS5487* and *HWGBS5600* was stable and observed in three of the four environments, explaining phenotypic variance from 7.8% to 14.2% and additive effects from 3.34 to 3.64. Except for QTL *Qfll*.*hww-5A*, additive effects for all the QTLs identified in this study were positive, suggesting the parent Harry conributes alleles to increase the FLL.

For FLW, a total of 4 additive effect QTL were detected on chromosomes 1A, 1B, 5A and 7A, and the phenotypic variance explained by individual QTL ranged from 5.21 to 16.5%. The additive effects (−1.01 to 1.04) of detected QTL was both positive and negative suggesting increasing alleles for flag leaf length were contributed by both the parents. The QTL detection in the four environments was highly variable. For instance, a single QTL was detected in the environments L15 and M16, whereas, 2 and 3 QTLs were detected in the environments L16 and M15. The *Qflw*.*hww-1B* was large-effect QTL detected only in one environment and accounted for 16.5% of the phenotypic variation for FLW. The *Qflw*.*hww-5A* locus flanked by markers *HWGBS3329* and *HWGBS3337* was stable, and large-effect QTL consistently detected in all the four environments and explained phenotypic variance from 9.95 to 10.9%. The additive effect for this QTL was negative, suggesting parent Wesley contributed alleles for increased FLW.

For FLA, a total of 10 additive effect QTL were detected on chromosomes 2D, 3A, 3B, 4B, 5A, 6A, 7A and 7D, and the phenotypic variance explained by individual QTL ranged from 7.0 to 15.0%. The additive effects of detected QTL was both positive and negative suggesting both parents contributed alleles to increase FLA. All the 10 QTL identified for FLA were different and detected only in one environment. There were three large effect QTL identified, *Qfla*.*hww-2D*.*a* (11.0% phenotypic variance explained), *Qfla*.*hww-5A* (15.02% PVE) and *Qfla*.*hw-7A* (14.5% PVE), whereas the rest of the QTL identified had small effects (7.2 to 9.6% PVE).

### QTL x environment interactions for FLL, FLW and FLA traits

QTL x environment interactions were detected for 22.2%, 16.6% and 25.2% of QTL for FLL, FLW and FLA respectively. Total phenotypic variance (PVE), phenotypic variance explained by additive effects (PVE (A)) and phenotypic variance explained by additive x environment effects (PVE (AxE)) is shown in Table 3. For FLL, QTL on 5A (*Qfll*.*hww-5A*) and 7A (*Qfll*.*hww-7A*.*b*) chromosome had strong QTL x environment interactions, which is evident by small LOD score for additive effects (LOD (A)) and high LOD score for additive by environment effects (LOD (AE)). Other QTLs detected for FLL were mainly specific to some of the environments with large LOD (A) and small LOD (AE). For FLW, out of 6 significant QTLs, only 1 QTL on 1B (*Qflw*.*hww-1B*) had strong QTL x environment interactions. Similarly for FLA, out of 8 significant QTLs, QTL on 2D (*Qfla*.*hww-2D*) and 5A (*Qfla*.*hww-5A*) had strong QTL x environment interactions, and the other 6 QTL were mainly specific to some of the environment.

**Table 3:** QTL x environment interactions affecting flag leaf length (FLL), flag leaf width (FLW) and flag leaf area (FLA) traits detected in four environments.

Trait	QTL	Chr^1^	Pos.^2^	Marker interval	LOD	LOD(A)^3^	LOD(AxE)^4^	PVE^5^	PVE(A)^6^	PVE(AxE)^7^
FLL	*Qfll*.*hww-6A*	6A	64	*HWGBS3922-HWGBS3920*	10.30	8.61	1.69	7.30	6.19	1.10
	*Qfll*.*hww-7A*.*a*	7A	78	*HWGBS5487-HWGBS5600*	9.50	6.94	2.57	9.73	4.91	4.82
	*Qfll*.*hww-2D*.*a*	2D	58	*HWGBS1918-HWGBS1863*	9.41	6.27	3.14	7.10	4.59	2.51
	*Qfll*.*hww-2D*.*b*	2D	13	*HWGBS1948-HWGBS1887*	8.01	5.20	2.82	6.02	3.77	2.25
	*Qfll*.*hww-4A*	4A	8	*HWGBS2903-HWGBS2830*	5.97	4.73	1.24	3.70	3.37	0.33
	*Qfll*.*hww-5A*	5A	28	*HWGBS3329-HWGBS3337*	5.45	0.15	5.30	3.33	0.10	3.23
	*Qfll*.*hww-7B*	7B	65	*HWGBS5851-HWGBS5846*	5.13	4.50	0.63	3.56	3.18	0.38
	*Qfll*.*hww-7A*.*b*	7A	75	*HWGBS5458-HWGBS5471*	5.06	0.59	4.47	1.94	0.42	1.52
	*Qfll*.*hww-7b*	7B	33	*HWGBS5874-HWGBS6009*	4.83	2.70	2.13	3.40	1.94	1.46
FLW	*Qflw*.*hww-1A*	1A	111	*HWGBS136-HWGBS115*	7.97	6.93	1.04	5.63	3.93	1.70
	*Qflw*.*hww-1B*	1B	66	*HWGBS799-HWGBS805*	14.88	4.05	10.83	5.43	2.42	3.00
	*Qflw*.*hww-3A*.*a*	3A	7	*HWGBS2052-HWGBS2055*	6.97	5.99	0.98	3.85	3.32	0.53
	*Qflw*.*hww-3A*.*b*	3A	165	*HWGBS2110-HWGBS2108*	6.63	6.25	0.38	4.43	3.56	0.87
	*Qflw*.*hww-5A*	5A	28	*HWGBS3329-HWGBS3337*	17.94	15.52	2.42	10.69	9.63	1.06
	*Qflw*.*hww-7A*	7A	87	*HWGBS5576-HWGBS5480*	7.18	5.06	2.12	3.09	2.90	0.19
FLA	*Qfla*.*hww-2D*.*a*	2D	27	*HWGBS1929-HWGBS1851*	6.28	3.64	2.64	4.86	2.58	2.28
	*Qfla*.*hww-2D*.*b*	2D	63	*HWGBS1860-HWGBS1994*	4.84	1.77	3.07	2.62	1.24	1.38
	*Qfla*.*hww-3A*	3A	0	*HWGBS2032-HWGBS2072*	7.85	7.56	0.30	5.69	5.29	0.40
	*Qfla*.*hww-3B*	3B	182	*HWGBS2422-HWGBS2280*	5.05	3.13	1.92	4.05	2.16	1.89
	*Qfla*.*hww-4B*	4B	33	*HWGBS3140-HWGBS3186*	5.92	5.02	0.91	4.88	3.57	1.31
	*Qfla*.*hww-5A*	5A	28	*HWGBS3329-HWGBS3337*	9.04	4.63	4.71	7.13	3.39	3.84
	*Qfla*.*hww-6A*	6A	72	*HWGBS3975-HWGBS3938*	6.78	6.22	0.57	5.26	4.43	0.83
	*Qfla*.*hww-7A*	7A	78	*HWGBS5487-HWGBS5600*	14.00	12.95	1.04	9.55	9.36	0.19

^1^Chromosome where the QTL was located. ^2^Chromosome position (cM) of the QTL. ^3^LOD score for additive effects. ^4^LOD score for additive by environment effects. ^5^Phenotypic variance explained by QTL. ^6^Phenotypic variance explained by additive effects. ^7^Phenotypic variance explained by additive x environment effects.

## Discussion

Integrating GBS and bi-parental mapping is becoming a powerful tool to develop high-density linkage maps, dissect the complex traits, and identify key genomic regions underlying these traits^30^. In this study, we used a GBS approach for genome-wide identification of SNPs and their utilization for the development of high-density linkage map in RIL population of bread wheat derived from highly different parents Harry (drought tolerant) and Wesley (drought susceptible)^22,23^. The purpose of developing RIL population was to determine genomic regions associated with agronomic traits in response to drought and adaptation across multiple rainfed environments. To dissect the complex traits and precisely map genomic regions high-density linkage maps are a prerequisite. A high-density linkage map was developed containing 3,641 markers distributed on 21 chromosomes. The strong collinearity of marker position and order with POPSEQ-based on high-density linkage map indicated the robustness of the constructed linkage map. The accuracy of linkage map was validated by mapping the genomic regions for two highly heritable traits of CC and CW to previously mapped regions in the bread wheat genome.

Initial map constructed (before error correction/filtering for low-quality markers) with 6,120 markers was 3.7 times the size of the final map and nearly double than the previously developed GBS based linkage maps in bread wheat^9,27,28^. Linkage map expansion is likely due to the cumulative effect of genotyping errors; every 1% error rate in a marker adds approximately two cM to the linkage map^14–16^. By removing genotyping errors and highly segregation distortion markers, the total length of final linkage map was reduced to 1,959 cM. Besides double recombinants, distorted loci may also impede precision of linkage mapping due to under- or over-estimation of recombination fractions^31,32^. The removal of segregation distortion markers reduces the genome coverage. In this study, significant segregation distortion markers were excluded from the map to obtain an accurate distance between markers in chromosomes. Linkage map expansion by a factor of 1.6, due to genotyping errors, in wheat using GBS data set has been recently reported by Saintenac *et al*.^16^. In other crops during the construction of linkage maps, linkage map expansion due to genotyping errors (double recombinants) also have been reported^33,34^. The total length of constructed linkage map (1,959 cM) from this study was lower than those reported in previous studies in wheat^9,27,35^, and is likely due to: (i) lower marker number, (ii) genetic constitution of mapping populations influencing rates of recombination^36^, and (iii) stringent filtering and, removing of potential genotyping errors and distorted loci.

The final linkage map constructed contained 3,641markers (684 bins) distributed across 26 LGs, the LGs from same chromosomes were combined to form 21 chromosomes that correspond to haploid chromosome number (n = 21) of bread wheat (See materials and methods for details). Combining the LGs from same chromosome results in gaps more than 30 cM but less than 50 cM. The discrepancy between haploid chromosome number and the number of LGs in wheat during linkage map construction is common^37–39^, and the discrepancy may be due to following reasons: (i) insufficient linkage between markers in different arms of the chromosomes, (ii) markers detected may not be evenly distributed across chromosomes and do not sufficiently cover the genome^40^, (iii) several areas may remain undetected due to polyploidy nature and large genome size (~16GB) of wheat^41^, and (iv) small population size^42^.

The 3,641 markers in final linkage map distributed across A, B and D wheat genome, with a maximum percentage of markers in B genome (55.8%) and minimum in the D genome (7.7%) (Table 1). The lower number of markers in the D genome as compared to A and B genome of wheat indicates that diversity in A and B genome is more and consistently lower in D genome which is accordance with the previous reports^35,39,43,44^. Lower density observed in D genome may also be because the two parental lines used to develop the bi-parental mapping population come out of the same breeding program, hence many chromosomal regions may be identical by descent. The marker density of 1.8 cM obtained in this study is comparable with or lower than the marker density of genetic maps constructed in wheat using the GBS approach^21,22,42–44^. In the present linkage map, several large gaps (>10 cM) exists; most of these gaps could be due to the removal of markers during the filtering process, however, for better coverage of map and to fill gaps additional markers must be added to the current map.

Segregation distortion is an important biological phenomenon that can cause the locus of interest and the flanking regions to deviate from the expected Mendelian ratio, thus forming segregation distorted regions^45,46^. In this study, 21.2% percent of mapped markers present on all chromosomes exhibited segregation distortion, and most of the segregation distortion markers tended to be clustered (forming segregation distorted regions) across the chromosomes The presence and clustering of distorted markers in all wheat chromosomes have been reported previously^27,31,32,47–49^. In this study, a total of 75 segregation distorted regions were identified on all chromosomes except chromosome 1D and 3A (Table 1). The occurrence of segregation distortion markers in clusters indicates that segregation distortion arise mainly due to biological factors rather than by technical problems^50^. The largest segregation distorted regions (with longer blocks, >30 markers) in increasing order were noticed on 1B, 6B, 6A, 7A, 3D and 3B with marker alleles mainly distorted towards male parent (Fig. 4). The largest segregation distorted regions on 1B and 6B in this study coincide with the previous studies in wheat^27,31,43,51^. The severe segregation distortion on 1B and 6B has been linked to the alien introduction (1B/1R translocation) and the presence of pollen killer gene *Ki* on 6B^52,53^. In the present study, segregation distortion on 1B, 6B and other chromosomes is unknown as there is no report of 1B/IR translocation and pollen killer gene in the parental lines Harry and Wesley^22,23^. Therefore, some other mechanisms like preferential pollination or abortion of male or female gametes or zygotes, may be involved in segregation distortion of these regions^54,55^.

For species with a reference genome sequence, markers can be ordered on the physical map. However, lacking high-quality reference genome in wheat, the POPSEQ (a reference map-based marker ordering method) has been used to map and order the GBS markers^38^. In this study, we compared the marker order of our map with POPSEQ v2.0-based on high-density linkage map developed by Chapman *et al*.^29^, to see how well linkage map is constructed. We found strong agreement in marker order of GBS markers across all 21 chromosomes (Fig. 5), indicating the robustness of our linkage map. Recently, application of POPSEQ for marker ordering in a hexaploid wheat RIL population (SynOpRIL) was demonstrated by Edae *et al*.^38^ and authors concluded that the POPSEQ-based reference map is a valuable resource that can be used to order GBS markers in hexaploid wheat.

The accuracy and reliability of a linkage map for QTL mapping was evaluated by locating the QTL for highly heritable traits of CC and CW. The underlying genomic regions responsible for CC and CW traits are known. The CC in hexaploid wheat is determined by a single dominant gene *Rg1* located towards the end of 1BS chromosome^56,57^. In the present study, we found a single major QTL (*Qcc*.*hww-1B*) for CC located towards the end of 1B chromosome (1BS based on GBS reference position of SNP marker). Similarly, in hexaploid wheat, CW is mainly controlled by two sets of genes: glaucousness loci (*W1* and *W2*) and non-glaucousness loci (*w1* and *w2*) located on 2BS and 2DS chromosomes^58,59^. QTL mapping performed for CW identified a single major QTL (*Qcw*.*hww-2D*) located on the 2D chromosome (2DS based on GBS reference position of SNP marker). Mapping of the highly heritable trait QTL of CC and CW exactly at same chromosome and genomic regions indicates that the map constructed is highly accurate and of high quality for genetic mapping and QTL identification. The quality and applicability of high-density linkage maps for QTL mapping have been demonstrated previously with highly heritable traits by various researchers in other crops^60–62^.

To demonstrate the applicability of linkage map for QTL mapping of quantitative traits, QTL mapping was performed for three flag leaf-related traits including FLL, FLW, and FLA. Most of the QTL for FLL, FLW and FLA identified in this study were reported by previous researchers on same chromosomes^18–21,63,64^. We also identified novel QTL for FLL and FLW on 1A, 1B, and 7A that were previously not reported. Of the 30 QTL detected in four environments (2 locations and two years) for FLL, FLW and FLA, one QTL each for FLL and FLW on chromosomes 7A (*Qfll-hww-7A*) and 5A (*Qflw-hww-5A*) were major stable QTL. The major stable QTL for FLL (*Qfll-hww-7A*) on 7A chromosome was identified the first time in this study. In wheat QTL for FLW on chromosome 5A has been reported by various researchers^19–21^. Recently, Xue *et al*.^65^ reported the fine mapping of a QTL (*Qflw*.*nau-5A*) for FLW on chromosome 5A. Further, most of the QTL identified in this study for all traits were minor QTL (explaining less than 10% of phenotypic variation) which confirms the polygenic control of flag leaf-related traits^18,19,66^. The significant amount of phenotypic variation that remains undetected in this study may be due to the following reasons, (i) few QTLs with subtle effects may fall below the significance threshold of detection^67^, (ii) due to epistatic interactions^67^, and (iii) environmental effects, population size, and experimental error^41^. The identification of major stable QTL identified in this study may be of immense value in marker-assisted selection (MAS) programs designed to improve wheat flag leaf size and yield potential in wheat breeding programs. Also, the stable major QTL identified in this study may provide a better platform for map-based cloning of underlying genes of these important QTLs.

In this study, co-located QTL (co-located QTLs either correspond to closely linked but distinct genes or to a single gene with pleiotropic effect) were found on chromosomes 2D, 5A, and 7A. Co-located QTL for flag leaf-related traits have been observed by previous researchers in wheat^18,19,21^. The co-located QTL on chromosome 5A in marker interval between *HWGBS3329-HWGBS3337* was involved in a FLL QTL identified in one environment, a FLW QTL identified in four environments and a FLA QTL detected in one environment (Table 2). Another co-located QTL were located on chromosomes 2D and 7A involved QTL controlling FLL and FLA. Co-localization may suggest pleiotropy or close linkages whereby a genomic region contains a cluster of QTLs that affect some traits^68^.

At the molecular level, the phenotypic plasticity (the ability of a genotype to produce multiple phenotypes in response to environment) arises from interactions between QTL and environments^69^. Understanding QTL x environment interactions will help to identify and breed stable genotypes across different environments which will provide a solid foundation for the genetic improvement of productivity^70,71^. The previous studies on QTL x environment interactions for flag leaf-related traits showed that QTL expressions varied across environments^18,19,72^. QTL x environment interactions for flag leaf-related traits was also evident in this study, and FLA, as compared to other traits, was effected more by the environment due to a higher number of interacting QTL and low heritability (Table 3 and Supplementary Table S2). In total, 23 QTL showed significant QTL x environment interactions, indicating genetic variation for phenotypic plasticity. Also, most of the QTL for FLL and FLW and all identified QTL for FLA were detected in single environments suggesting differential expression pattern of QTL in response to different environments^18,72^. The effect of environment on QTL expression demonstrate that QTL x environment interactions is an important component of the genotypic variance and can play an immense role in improving flag leaf size in future wheat breeding endeavors. From the breeding point of view, a QTL identified can be used in broad range of environments or specific environments depending upon the absence/small or presence of QTL x environment interactions.

In summary, GBS platform was used for genome-wide identification of SNPs, and a high-density linkage map was constructed. The high-density linkage map developed would provide the basis for the next phase of research such as QTL and fine mapping of quantitative traits in Harry x Wesley derived RIL population, comparative mapping, map-based cloning, and in marker-assisted breeding endeavors in wheat.

## Materials and Methods

### Plant material

A population of 204 RILs (F_6:8_) obtained from the cross between ‘Harry’ x ‘Wesley’ through single seed decent method was used for genotyping. Harry is white chaffed hard red winter wheat known for its superior adaptation to dryland systems in Western Nebraska^22^. Wesley a bronze chaffed hard red winter wheat is drought-sensitive cultivar better suited for irrigated or high moisture production systems^23^.

### Experimental design and phenotypic evaluation

Field trials were conducted during 2014-2015 (hereafter referred as the year 2015) and 2015-2016 (hereafter referred as the year 2016) growing seasons at the University of Nebraska, USA research farms Lincoln (N 40° 30.822 W 73° 21.508) and Mead (N 41° 08.782 W 096° 29.985), resulting in a total of four environments. The 204 RILs along with three checks (two parental lines: Harry and Wesley and ‘Overland’) were evaluated in an augmented randomized complete block design (RCBD) with 12 incomplete blocks, and each block contained 17 RILs (un-replicated) plus three checks (replicated in each block) for a total of 20 lines per incomplete block. The experimental unit was a four-row plot of 3 m length and 1.2 m width. The field management was followed by the standard agricultural practices for all the locations, and each plot was sprayed with fungicide to control the fungal diseases. Phenotyping was done for chaff color (CC), leaf cuticular wax (CW), flag leaf length (FLL), flag leaf breadth (FLB), and flag leaf width (FLW). The CC and CW data was collected during the year 2015 at locations Lincoln and Mead. FLL, FLW, and FLA were collected during two years (2015 and 2016) at locations Lincoln and Mead. The CC was recorded as dark (score of 1) or white (score of 0) for every line/plot. The CW phenotype was visualized and recorded as per Lu *et al*.^58^. The FLL (measured in centimeters from leaf collar to the tip) and FLW (measured in centimeters on the widest part of the leaf) were measured on ten randomly selected plants from the main tiller of each plant. The derived trait FLA (cm^2^) was calculated as FLA = FLL × FLW × 0.8^18^. The four environments Lincoln 2015, Lincoln 2016, Mead 2015 and Mead 2016 hereafter will be called as L15, L16, M15, and M16.

### Statistical analysis of phenotypic data

Descriptive statistics and analysis of variance (ANOVA) was conducted using SAS 9.2 software^73^. Descriptive statistics and normality test were performed with PROC UNIVARIATE procedure, and ANOVA was performed with PROC MIXED procedure, where checks, years and locations were treated as fixed effects, and lines, line × location, line x year, blocks nested in location and year were treated as random effects.

For QTL mapping Best Linear Unbiased Predictors (BLUPs) were estimated from the random effect of genotypes using ASReml-R package^74^ in statistical software R version 3.1.3^26^ (Supplementary Data S2). For estimation of entry-mean based heritability (*h* ^*2*^) variance components were obtained by the restricted maximum likelihood (REML) method assuming a full random model in ASReml. The formula used to estimate heritability is:

$${h}^{2}=\frac{{\sigma }_{g}^{2}}{{\sigma }_{g}^{2}+\frac{{\sigma }_{ge}^{2}}{e}+\frac{{\sigma }_{e}^{2}}{re}}$$

where, ${{\rm{\sigma }}}_{{\rm{g}}}^{{\rm{2}}}$ is variance component for genotypes, ${{\rm{\sigma }}}_{{\rm{ge}}}^{2}$ variance component for genotype x environment, ${{\rm{\sigma }}}_{e}^{2}$ variance component for error; r is a number of replications and e is a number of environments.

### GBS library construction, genotyping and SNP calling

DNA was isolated and purified for all the 204 RILs (F_6_:_8_) plus two parents (Harry and Wesley) from the leaves of young 2-3 seedlings (two-week old seedlings) using BioSprint 96 DNA Plant Kits (Qiagen). GBS was performed according to the protocol described by Poland *et al*.^9^. Briefly, GBS libraries were constructed in 192-plex, and genomic DNA was digested with the restriction enzymes *PstI* and *MspI*. Pooled libraries were sequenced using Illumina, Inc. NGS platforms. Raw data FASTQ file containing sequence reads were processed for SNP identification using GBS analysis pipeline implemented in TASSEL v4.0^75^ using default parameters (except the minimum count of reads for a GBS tag to be output was changed from the default value of 1 to 5). Chinese Spring genome assembly from IWGSC was used as reference genome^76^. Raw sequence data of 204 RILs along with additional ~3,000 wheat breeding lines in Nebraska wheat breeding program were analyzed together. The purpose to perform the combined analysis of RILs along with additional wheat lines was to increase the genome coverage and read depth for SNP discovery in RILs^77,78^. Initially, over 200,000 SNPs were generated for all lines including 204 RILs. The average accuracy of SNP calling tested using the pair-wise comparison of check cultivars genotyped in multiple years was >95%^79^. SNPs with the maximum missing percentage of less than 80% were retained and subsequently processed through the imputation algorithm using Beagle v. 4^80^, with default parameters, and the genotype calls were successively imputed. Further filtering of SNPs by applying filtering relevant to only 204 RILs (retaining SNPs with minor allele frequency greater than 0.2 and allelic R^2^ estimated in imputation algorithm Beagle v.4 greater than 0.5), resulted in 8,505 SNPs. Subsequently, ABH-plugin in TASSEL was applied to convert SNP calls from a nucleotide-based format to a parent-based format, and the total number of SNPs retained for linkage mapping were 6,120 SNPs.

### Linkage map construction

A linkage map was constructed in series of steps, and before linkage map construction all heterozygous calls were scored as missing data. In the first step of linkage mapping, BIN tool algorithm implemented in ICI mapping software v4.1^81^ was used, and binning of all 6,120 SNPs was done based on their pattern of segregation^39^. A “bin” means a group of markers with an identical segregation pattern (zero recombination among markers) that is separated from adjacent bins by a single recombination event^8^. After binning, markers were grouped using logarithm of the odds (LOD) threshold values ≥4.5. Linkage groups (LGs) were assigned using the genomic position of SNP markers determined during the SNP calling. LGs from the same chromosome were merged, and LGs with fewer than five markers were deemed to be unlinked and dropped from further construction. RECORD (Recombination Counting and ORDering) algorithm was used to order all the markers within LGs. Recombination frequencies between markers were converted into centiMorgans using the Kosambi mapping function. The initial linkage map constructed was implemented in R/qtl^24^ and R/ASMap^25^ packages available in R Statistical Computing Environment^26^ to inspect the markers for duplicate lines, segregation distortion, switched alleles, single and double cross-overs (genotyping errors) using the appropriate functions. The details and R scripts used to inspect and remove low-quality markers and genotypes is given in Supplementary Methods.

In the second step of linkage mapping i.e., after error correction and dropping of low-quality markers and genotypes, genotypic data from 194 lines with 3,641 filtered SNP markers was used for final linkage map construction in ICiMapping v4.1^81^. LGs were determined with LOD threshold >4.5, and 26 LGs representing all 21 wheat chromosomes were identified. Chromosomes 2A, 5A, 5B, 6A and 7A that split into two linkage groups, were re-formed into single linkage group at lower LOD score. The ordering of 3,641 SNP markers distributed over 21 chromosomes was performed using RECORD algorithm, and rippling to fine tune the marker order was performed by sum of adjacent recombination fractions (SARF) with a window size of 7 as rippling criteria. Genetic distances of SNP markers based on recombination rate were converted using the Kosambi mapping function. Finally, the best marker order with the shortest linkage map distance was selected, and a linkage map was constructed and plotted in R/qtl using command *plotmap*.

For various plot visualizations, ggplot2 package in R software was used, and all R-scripts used is provided in Supplementary Methods. Further, the data used to draw all the plots is provided in Supplementary Data 3.

### QTL mapping

QTL mapping was performed using BIP functionality in QTL IciMapping v4.1^81^. Inclusive Composite Interval Mapping of Additive (ICIM-ADD) function in QTL IciMapping was selected as mapping method to detect additive QTL. The mapping parameters step for ICIM-ADD was set at 1.0 cM, and a probability of 0.05 in stepwise regression was selected for each mapping method. The LOD threshold for QTL detection was determined by 1000 permutation test analyses using a Type I error set at P < 0.05. QTL x environment interactions were detected using the Multi- Environment Trials (MET) functionality^82^. Additive x environment (AE) interactions were detected using 1.0 cM steps in scanning, a probability of 0.001 for stepwise regression. Significant AE interactions were claimed at P < 0.05 (LOD = 3). The MET functionality in ICi Mapping software which uses multi-environment data simultaneously not only assessed the QTL x environment interactions but also detects additional QTLs which are not identified in single environment analysis. This procedure is advantageous because it provided more precise and reliable position of QTLs identified in single environment analysis^82^. QTL for all traits were designated according to standard nomenclature^56^. We defined a major QTL as a QTL with a LOD value score >3 and a phenotypic variance contribution of ~10% or more in at least two or more than two environments. And we defined a stable QTL as a QTL that showed significance in at least three of the four environments (L15, L16, M15 and M16).

### Data availability

The GBS data of 204 RILs plus two parents (Harry and Wesley) have been submitted to the NCBI Sequence Read Archive with the BioProject ID: PRJNA381154 (https://www.ncbi.nlm.nih.gov/bioproject/PRJNA362959). Marker data before filtering and after filtering is given in Supplementary Data S1. BLUPs used for QTL mapping of flag leaf traits is provided in Supplementary Data S2. The data to create all plots in this paper is given in Supplementary Data S3. All codes to filter the low-quality markers and plot various figures in R/qtl and R/ASMap is given in Supplementary Methods.

## Electronic supplementary material

Supplementary Information

Dataset 1

Dataset2

Dataset3
