# Supplementary material for: Genotyping-by-Sequencing Derived High-Density Linkage Map and its Application to QTL Mapping of Flag Leaf Traits in Bread Wheat
Source: Sci Rep. 2017 Nov 27;7:16394. doi: 10.1038/s41598-017-16006-z (PMC5703991; doi:10.1038/s41598-017-16006-z)
Supplement: Supplementary file 1 — Supplementary Information [file 41598_2017_16006_MOESM1_ESM.pdf]

Supplementary Information

Genotyping-by-Sequencing Derived High-Density Linkage Map and its Application to QTL Mapping of Flag Leaf Traits in Bread Wheat

Waseem Hussain[1], P. Stephen. Baenziger[1]*, Vikas Belamkar[1], Mary J. Guttieri[2], Jorge P. Venegas[1], Amanda Easterly[1], Ahmed Sallam[3], Jesse Poland[4]

[1]Department of Agronomy and Horticulture, University of Nebraska-Lincoln, NE 68583, USA

[2]USDA, Agricultural Research Service, Center for Grain and Animal Health Research, Hard Winter Wheat Genetics Research Unit, 1515 College Avenue, Manhattan, KS 66502

[3]Department of Genetics, Faculty of Agriculture, Assiut University, 71526-Assiut, Egypt

[4]Wheat Genetics Resource Center, Department of Plant Pathology, Kansas State University, Manhattan, KS 66506, USA

*pbaenziger1@unl.edu
Office: 402-472-1538
Fax: 402-472-7904

Supplementary Tables

Table S1. Phenotypic summary of flag leaf length (FLL), flag leaf width (FLW) and flag lead area (FLA) evaluated at four environments (2 years and 2 locations).

		**Parents**		**RILs**				
Environments	Traits	Harry	Wesley	Mean	Min	Max	SD[1]	CV[2]
L15	FLL	20.06	19.02	20.8	14.92	25.8	2.28	10.9
	FLW	1.56	1.53	1.58	1.35	1.90	0.13	8.06
	FLA	25.92	25.64	26.31	16.80	39.40	4.00	15.24
L16	FLL	20.50	18.30	20.30	14.48	27.73	2.88	14.16
	FLW	1.50	1.41	1.57	1.30	1.90	0.14	8.39
	FLA	24.68	20.56	25.49	16.14	37.58	4.55	17.82
M15	FLL	24.61	22.44	22.59	17.41	29.39	2.18	9.67
	FLW	1.72	1.60	1.62	1.20	2.26	0.18	10.84
	FLA	33.79	28.9	29.23	18.78	47.95	5.40	18.42
M16	FLL	25.90	23.70	25.24	20.00	31.34	2.20	8.72
	FLW	1.60	1.53	1.58	1.23	2.07	0.19	12.40
	FLA	33.22	29.21	32.13	21.76	48.46	5.25	16.34
Average	FLL	22.67	20.86	22.26	16.70	28.50	2.38	10.86
	FLW	1.59	1.52	1.58	1.27	2.03	0.16	9.92
	FLA	29.40	24.77	27.5	18.37	43.30	4.8	16.95

[1]Standard deviation
[2]Coefficient of variation

Table S2. Pooled analysis of variance for flag leaf length (FLL), flag leaf width (FLW) and flag leaf area (FLA) across four environments (2 years and 2 locations)

Traits ➡		FLL		FLW		FLA	
Source ⬇	DF	MS	F Value	MS	F Value	MS	F Value
Check	2	86.84	39.14**	0.18	15.44**	369.65	38.29**
Lines	203	14.71	3.69**	0.06	8.02**	58.30	5.06**
Block(location*year)	44	2.58	1.17^{ns}	0.012	0.45^{ns}	11.40	0.2^{ns}
Year	1	7.68	2.97*	0.20	16.37**	18.73	1.64^{ns}
Location	1	475.66	183.82**	0.33	26.47**	1357.46	119.05**
Year*check	2	16.18	7.29**	0.016	0.68^{ns}	37.77	3.91*
Year*lines	203	2.59	1.53**	0.009	1.03^{ns}	8.62	1.15*
Location*check	2	13.19	5.95**	0.008	0.65^{ns}	47.15	4.88**
Location*lines	203	3.08	1.82**	0.007	0.84^{ns}	10.41	1.38*
Location*year*lines	203	1.70	0.77^{ns}	0.009	0.77^{ns}	7.53	0.78^{ns}
Location*year	1	48.38	18.70**	0.0014	0.01^{ns}	64.96	5.70*
Residual	91	2.21		0.012		9.65	
Heritability		74%		78%		72%	

**significant at 1% level; MS= Mean square; DF=Degrees of freedom

*significant at 5% level

^{ns} non-significant

Supplementary Figures

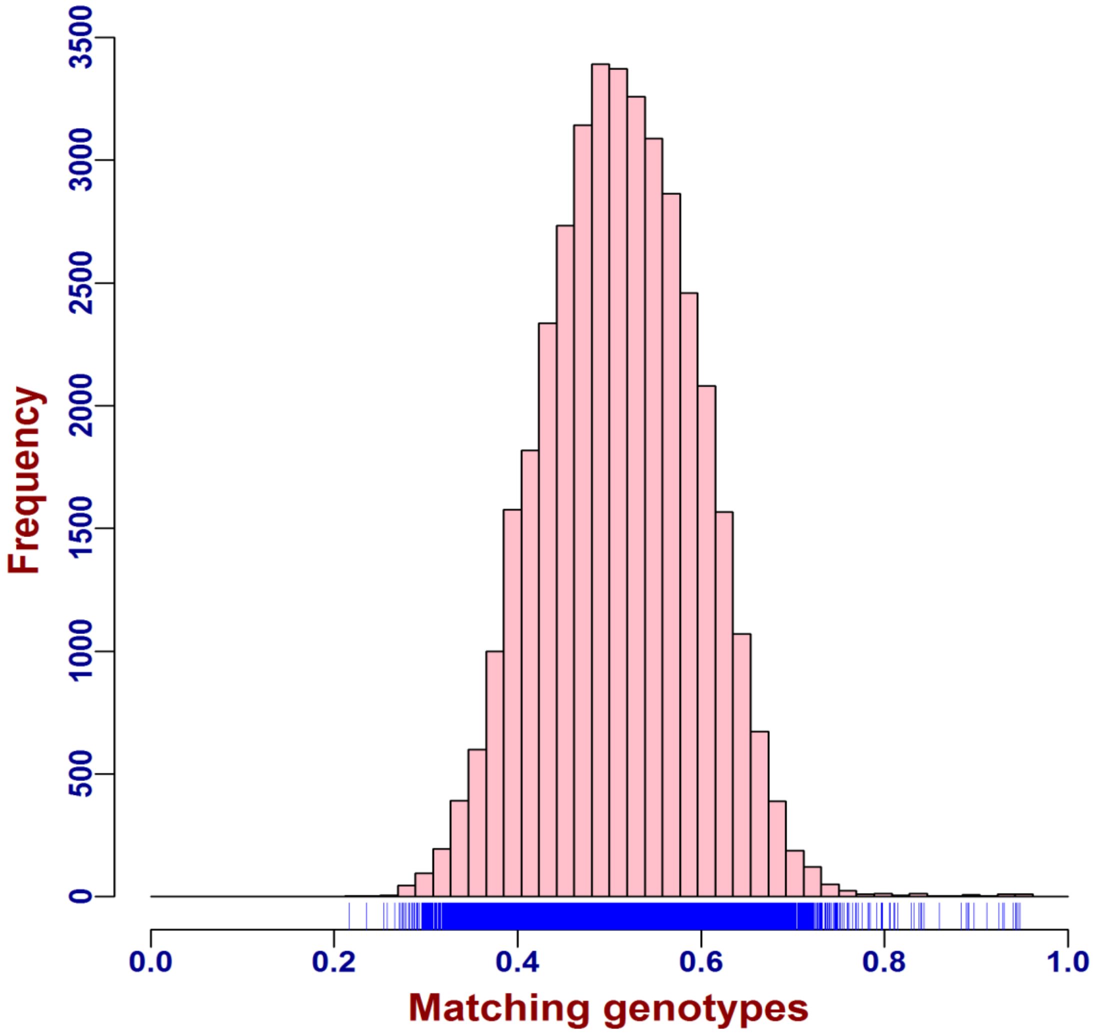

Figure S1. Proportion or number of matching recombinant inbred lines sharing same genotypic information.

(a)

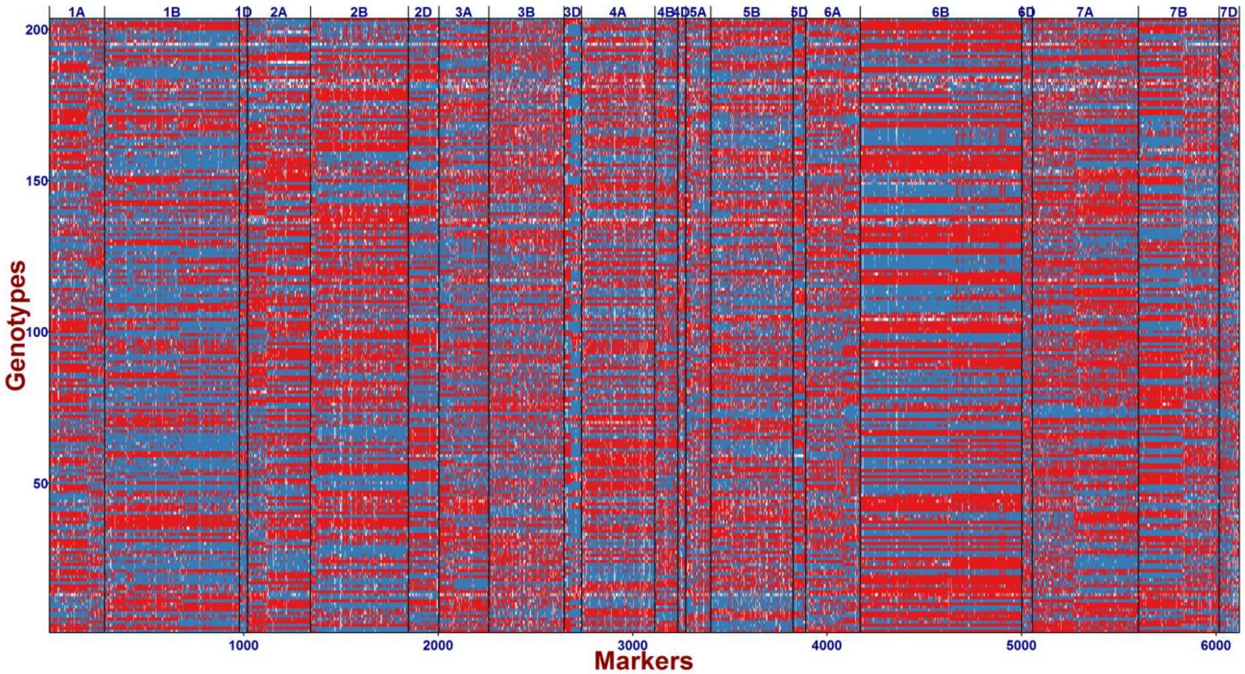

(b)

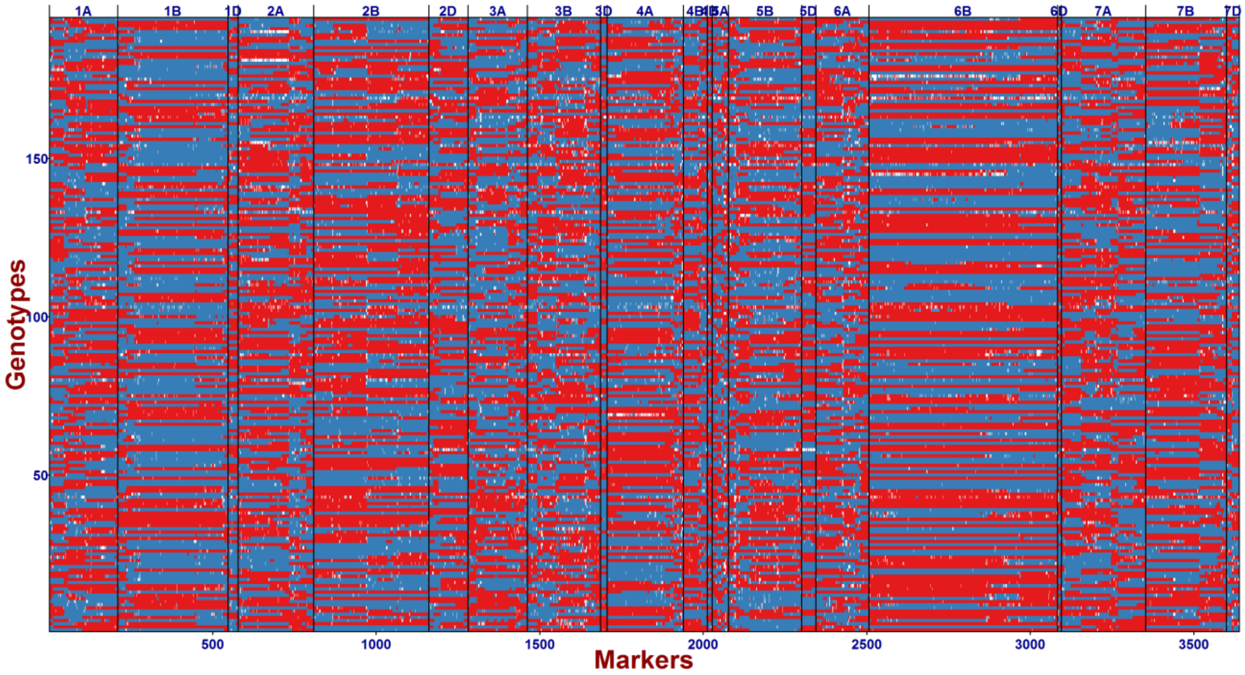

Figure S2. Graphical representation of genotypes on 21 chromosomes (a) before correction and (b) after correction. The red color represents alleles from parent A (Harry), blue color represents allele from parent B (Wesley), and white color represents missing information. Black vertical lines indicate the boundaries between chromosomes. Recombination blocks are clearly defined in (b) as compared to (a).

(a)

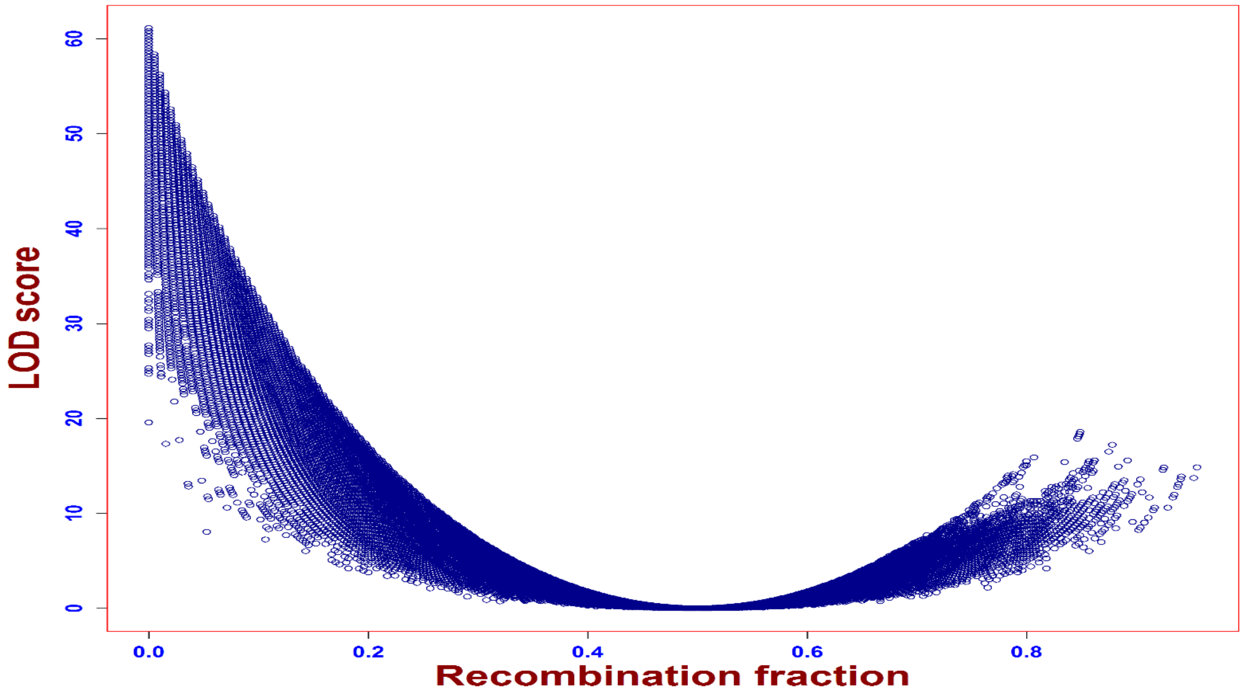

(b)

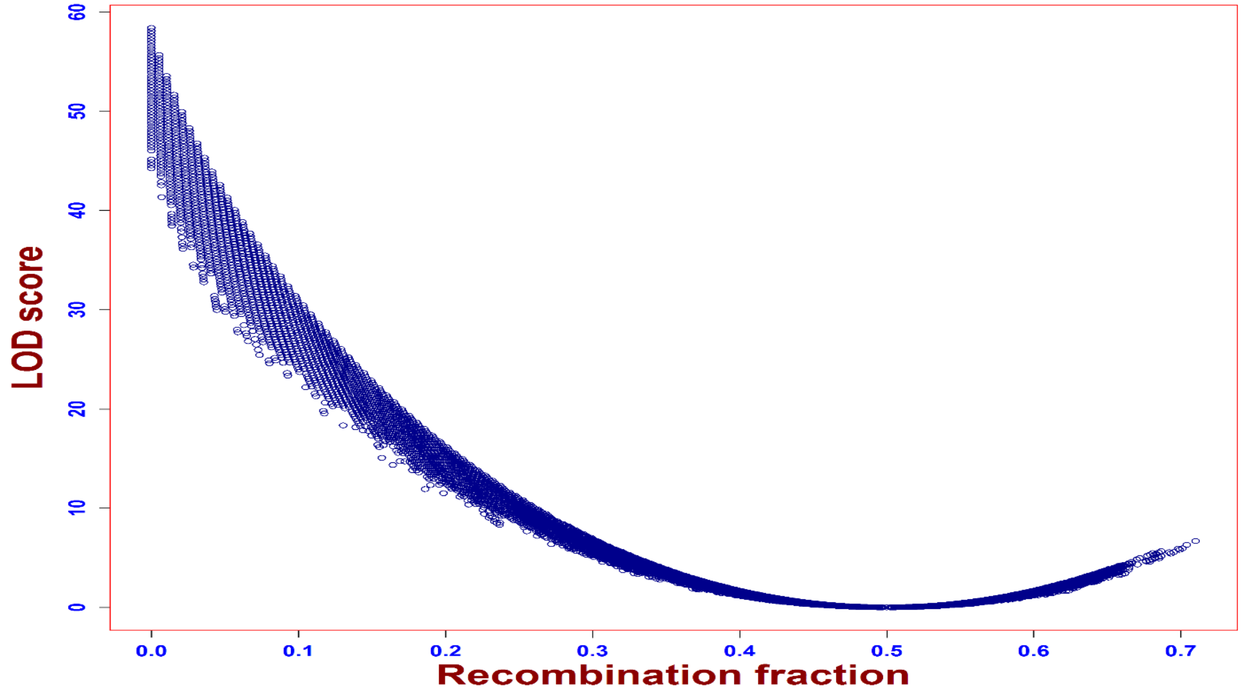

Figure S3. Pairwise recombination fraction of all marker pairs (a) before correction and (b) after correction. Higher recombination values and LOD score in uncorrected data set indicates the potential problem in markers.

(a)

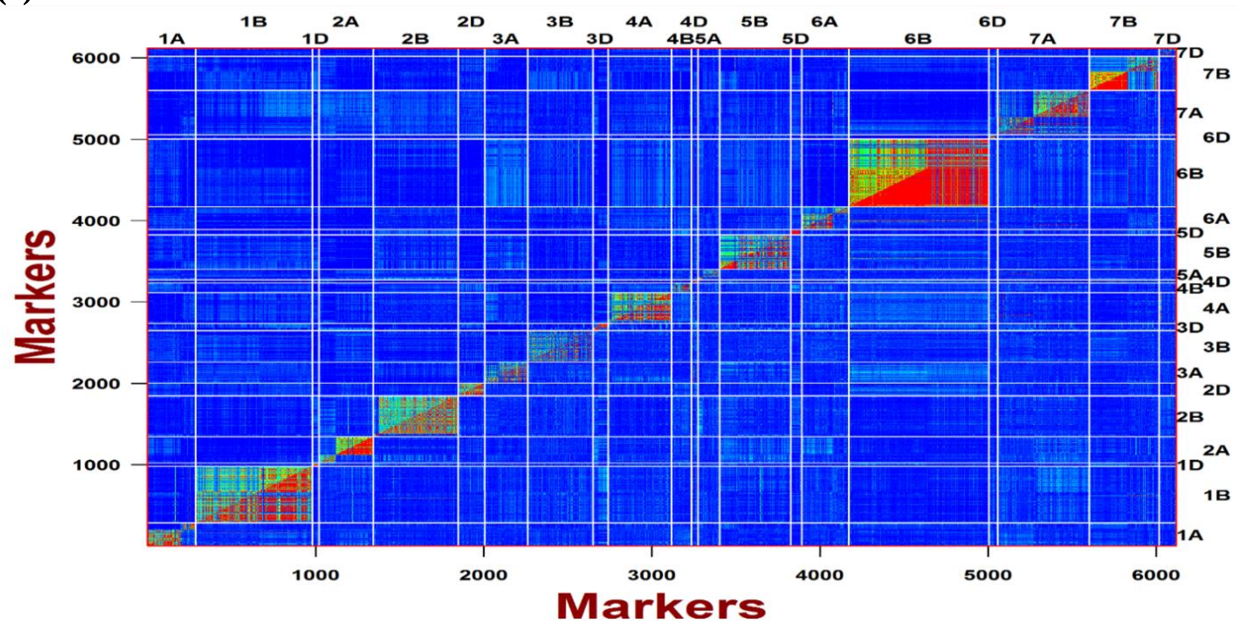

(b)

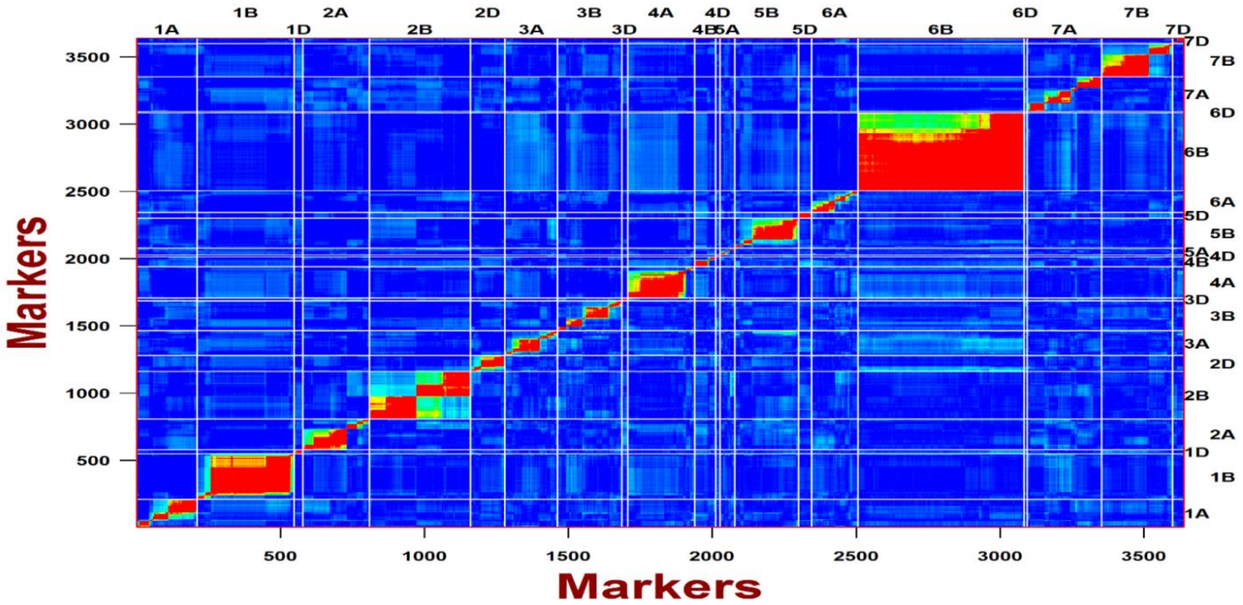

Figure S4. Heat map of pairwise recombination fractions (above diagonal) and LOD score (below diagonal) for all marker combinations (a) before correction (b) after correction. Low estimates of recombination fraction and high LOD score are shaded by red, while blue color represents converse. Strong recombination fraction and LOD score along diagonal and lack of in the off-diagonal in (b) show the robustness of developed linkage map. There are few number of red points in the lower right in (a), indicating a potential problem of linkage between markers on different chromosomes. Also blue color in the lower left triangle in (a) points markers on same chromosomes show weak or zero linkage which is unrealistic suggesting problems with the order of markers within the same chromosome in (a).

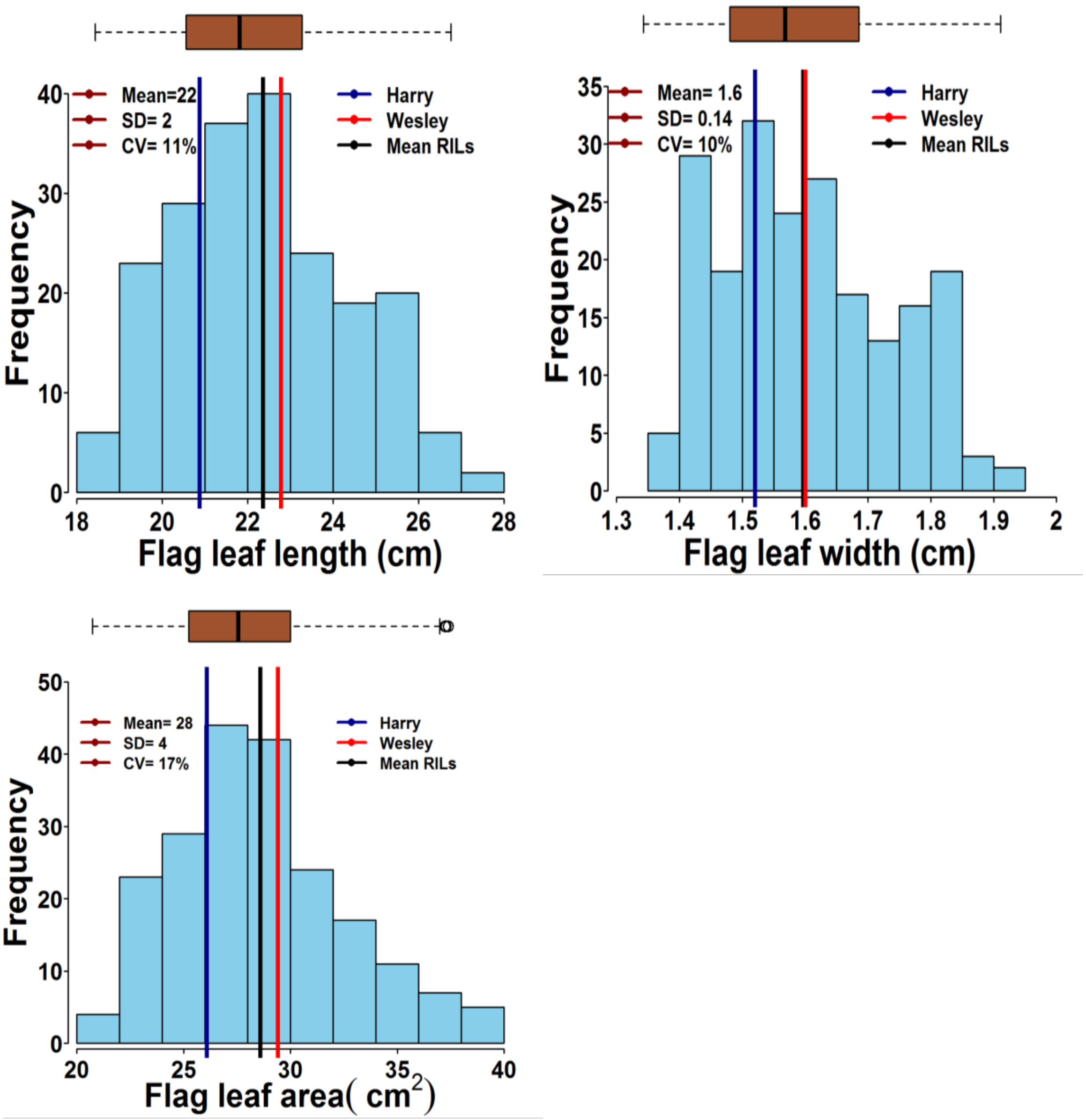

Figure S5. Frequency distribution for flag leaf length (FLL), flag leaf width (FLW) and flag leaf area (FLA) evaluated over four environments (2 years and 2 locations). Vertical lines indicate the mean of the parental genotypes Harry (blue line) and Wesley (red line), and RILs (black line). Mean of RILs, standard deviation (SD) and coefficient of variation (CV) are also show on top left corner of each figure.

Supplementary Methods

The R packages R/qtl[1,] and R/ASMap[2] was used to inspect the genotypic data and remove the low-quality markers and genotypes to ensure that high-quality markers and genotypes are being used to construct a final linkage map. The initial linkage map constructed in ICiMapping software v4.1[3] was organized in comma delimited file (*.csv) and read in R/qtl using command *read.cross*.

Markers or genotypes with a high proportion of missing information could indicate problems with the linkage map. However, no issues with missing data (<15%) in our data file as it was imputed, missing data only comes from heterozygous calls that were treated as missing.

Function *comparegeno* in R/qtl was used to compare the genotypes for all pairs of lines to identify duplicate genotypes (Supplementary Fig. S1). The function *geno.image* was used to visualize and plot the genotypes for 204 RILs (Supplementary Fig. S2 (a))

Significant distorted markers (Chi-square Test, $p < 0.01$) that deviate from expected 1:1 allelic ratio as assumed in bi-parental populations were identified and dropped using *geno.table* and *drop.markers* commands in R/qtl.

Pairwise marker linkages were studied to look for switched alleles. Alleles will be considered switched (A ↔ B) if they are tightly associated with other markers, but with recombination fractions (RFs) well above 0.5. To check for switched alleles, *checkAlleles* function was used in R/qtl, and no apparently problem was found in genotypic data. This was also confirmed by estimating RF between each marker using function *est.rf()* and plotted against LOD score of each marker (Supplementary Fig. S3 (a)).

Heat map between pairwise RF between markers as well as LOD score (Higher LOD indicate strong linkage between markers) is one of the useful visual diagnostic tool to check how well a linkage map is ordered across the linkage groups (LGs). Using the function *est.rf* () and *plotRF()* heat map was plotted (Supplementary S4(a)).

Further, for efficient linkage map construction and diagnosis, R/ASMap package was used to check for genotyping errors or elevated number of double recombinants. For the calculation and profiling of statistics (number of single crossovers and number of double crossovers) for genotypes, functions *statGen()* and *profileGen()* were used in R/ASMap. Similarly, *statMark()* and *profileMark()* functions were used for the calculation and profiling of statistics (double cross overs and allele proportions) associated with the markers. Genotypes and markers with inflated single

crossovers and double crossovers were dropped using *drop.markers* and *subset* functions. In total 1066 markers and 10 RIL genotypes were removed from the genotypic data due to inflated number of double recombinants. The number of high-quality SNP markers retained after error corrections/filtering were 3641 distributed over 21 LGs and these markers were used for final linkage map construction.

After error correction and construction of final linkage map, all 194 genotypes were again visualized using function *geno.image* in R/qtl (Supplementary Fig. S2 (b)). Genotypes were also visualized in Excel, and each allele was color coded using conditional formatting in Excel-2013 to display the data and check for any double recombinants. The final linkage map data set in Supplementary Data S1 is color coded using conditional formatting in Excel. RFs of all marker pairs and heat map of pairwise recombination fractions and LOD score for all marker combinations were also plotted and is given in Supplementary Fig. S3 (b) and Supplementary Fig S4 (b).

Note: We follow the guidelines outlined in R/qtl (http://www.rqtl.org/tutorials/geneticmaps.pdf) and R/ASMap (https://cran.rproject.org/web/packages/ASMap/vignettes/asmapvignette.pdf) tutorials to perform all the linkage map analysis and data corrections. The R scripts used are given below:

R scripts used for filtering/error corrections and plots

```
# Set working directory
    setwd("C:/Users/wasim/BoxSync/DataAnalysis_ALL/GenotypicDataanalysis/LinkageMa
    pping/Rqtl")
    rm(list=ls(all=TRUE)) # Clears out your prior workspace.
#Loading packages into R
    library(qtl)
    library(ASMap)
#Loading data from working directory
    HW<-read.cross("csv",file="Raw_GBS_HWdata_6120.csv",na.strings=c("-
    "),genotypes=c("A","B"), alleles=c('A','B'), crosstype="riself", estimate.map=FALSE)
    summary(HW)
    jittermap(HW)
#plot genotypic data using geno.image and save it
     tiff("Geno.image.tiff",width =10, height =8, units = 'in', res =300, compression="lzw)
# setting graphical parameters.
    par(cex.axis=1,  cex.lab=1.5,font.axis=2,font.lab=2,col.axis='darkblue',col.lab="darkred")
    par(mgp=c(2.3,0.8,0))
    par(mar=c(4,4,4,1))
# plot before error correction (BC)
```

```
plot_BC<-
geno.image(HW,alternate.chrid=TRUE,main="",xlab="Markers",ylab="Genotypes")
dev.off()
```

#Note: No issues with missing data (<15%) in our data file as it was imputed, missing data only comes from heterozygous calls that were treated as missing. However, the codes to check for missing data is given below

```
# check for mising data.
plotMissing(HW)
#plot missing by markers(ntyped) and individuals(nmissing)
par(mfrow=c(1,2), las=1)
plot(ntyped(HW), ylab="No. typed markers", main="No. genotypes by individual")
plot(ntyped(HW, "mar"), ylab="No. typed individuals",main="No. genotypes by marker")
```

#Check for duplicate individuals with high proportion of matching allelic information between them.

```
# genotypes that share a proportion of their alleles>95%
HW_duplicate <- genClones(HW1, tol = 0.95, id="Genotype")
HW2<- fixClones(HW1, HW_duplicate$cgd, consensus = TRUE)
# to obtain a single consensus genotype.
summary(HW2)
# Save as .csv file
write.cross(HW2, file="HWGBS_dropingduplicates.csv")
# Histogram of number of matching genotypes be plotted in R/qtl
tiff("Comparing_genotypes.tiff", units="in", width =6, height =6, bg="white", res=300,
compression="lzw")
cg <- comparegeno(HW2)
par(mar=c(3,3,1.2,1.2), mgp=c(1.8,0.4,0))
hist(cg, breaks=seq(0, 1, len=53), main="",xlab="Matching
genotypes",ylab="Frequency", col="pink", col.axis="darkblue", cex.axis=1, cex.lab=1.3,
col.lab="darkred",font.axis=2, font.lab=2)
rug(cg, col="blue")
dev.off()
#Check markers for excessive segregation distortion.
gt <- geno.table(HW2)
gt[gt$P.value < 0.01,]
gt[gt$P.value < 0.01/totmar(HW2),]
# drop significant distorted markers
todrop <- rownames(gt[gt$P.value < 0.01,])
write.csv(todrop, file="SD_markers.csv")
HW3<-drop.markers(HW2,todrop)
summary(HW3)
write.cross(HW3, file="HW_distorted_markers.csv")
#check markers for switched alleles
checkAlleles(HW3, threshold=3) # No apparent problem found
#Estimate and plot recombination fractions of all the markers
rf <- pull.rf(HW3)
lod <- pull.rf(HW3, what="lod")
```

```
tiff("Recombination_fractionBefore.tiff", width =12, height =10, units="in", res=300, compression="lzw")
par(mar=c(4,5,2,2))
par(cex.axis=1.3,cex.lab=2.4,font.axis=2,font.lab=2,col="red",col.axis="blue", col.lab="darkred")
plot(as.numeric(rf), as.numeric(lod), xlab="Recombination fraction", ylab="LOD score", col="darkblue")
dev.off()
# Heatmap plot for visual diagnosis between recombination fractions and LOD score
r<- est.rf(HW3)
tiff("HeatMap_beforecorrection.tiff", width =7.5, height =6, units="in", res=300, compression="lzw")
par(mar=c(4.6, 4.8,2,1.8))
par(mgp=c(2.6,0.8,0))
par(cex.axis=0.8, cex.lab=2, font.axis=2, font.lab=2, col="red",col.axis="black", col.lab="darkred")
plot<-plotRF(r, alternate.chrid=TRUE, main="")
dev.off()
# Marker profile and statistics in R/ASMaplibrary for checking genotyping errors
# Linkage map statistics across the markers
stat_markers<-statMark(HW3, stat.type = c("marker"), map.function = "kosambi")
write.csv(stat_markers, file="stat_markers.csv") # Save the marker statistics file
# visualize marker profile showing segregation distortion (seg.dist), double crossovers(dxo), estimated recombination fraction (erf), and LOD score  (lod) for each marker
profileMark(HW3, stat.type = c("seg.dist", "dxo", "erf", "lod"), id = "Genotype", layout = c(1, 4), type = "l", cex=2.5, cex.axis=2, cex.lab=2)
#Dropping the markers with elevated number of dxo using drop.markers function
HW4 <- markernames(HW3,)[statMark(HW3, stat.type ="marker")$marker$dxo>10]
HW4<-drop.markers(HW3, HW4)
Summary (HW4)
# Linkage map statistics across the genotypes
stat_genotype<- statGen(HW4, bychr = FALSE, stat.type = c("dxo"))
write.csv(stat_genotype, file="stat_genotype_dxo.csv")
# visualize genotype profile showing number double crossovers (dxo) and missing proportion (miss) in each genotype
profileGen(HW4, bychr = FALSE, stat.type = "dxo", id ="Genotype", lty = 20)
# Genotypes with elevated number of dxo were removed using subset() command
HW5<- subset(HW4, ind = stat_genotype$dxo<60)
# save the Data file for final linkage map construction
write.cross(HW5, file="HW5.csv")
```

R scripts used to plot distribution of bins (Fig. 2)

```
rm(list=ls) # clears prior work space
setwd("C:/Users/whussain2/BoxSync/DataAnalysis_ALL/GenotypicDataanalysis/LInkage Mapping/Rqtl/Bin_plot/ByGroup") # set working directory
library(ggplot2) # load package
```

Read the data files in .csv format

```
BIN_A<-read.csv(file="BINS_GROUPA.csv", header=T)
BIN_B<-read.csv(file="BINS_GROUPB.csv", header=T)
BIN_D<-read.csv(file="BINS_GROUPD.csv", header=T)
str(BIN_A)
```

#Genome A

```
BIN_A<-ggplot(BIN_A, aes(x=Position))+geom_bar(color="brown2", width=.3)+
theme_bw()+ # set theme background
facet_grid(.~Chromosome,scales = "free", space = "free")+ # Grids
geom_rug(sides="b", col="black", size=0.6)+ # add rugs to axis
labs(title = "", x = "Position of bin (cM)", y = "Markers per bin")+ # add labels to axis
theme (plot.title = element_text(color="black", size=14, face="bold"), # add tile and size
# add axis title, text and adjust size
axis.title.x = element_text(color="black", size=16, face="bold"),
axis.title.y = element_text(color="black", size=16, face="bold"))+
theme(axis.text = element_text(colour = "black"))+
theme(axis.text= element_text(face = "bold", color = "black", size = 11))+
theme(legend.title = element_text(colour="black", size=12, face="bold"),
# add legends, and text and theme for strips
legend.text = element_text(colour="grey0", size=11, face="bold"))+
theme(strip.text.x = element_text(size = 12,face="bold",colour = "black"))+
theme(strip.background = element_rect(fill = "lightblue", color = "black", size=1.5))
```

# Genome B

```
str(BIN_B)
BIN_B<-ggplot(BIN_B, aes(x=Position))+geom_bar(color="brown2", width=.3)+
theme_bw()+
facet_grid(.~Chromosome,scales = "free", space = "free")+
geom_rug(sides="b", col="black", size=0.6)+
labs(title = "", x = "Position of bin (cM)", y = "Markers per bin")+
theme (plot.title = element_text(color="black", size=14, face="bold"),
axis.title.x = element_text(color="black", size=16, face="bold"),
axis.title.y = element_text(color="black", size=16, face="bold"))+
theme(axis.text = element_text(colour = "black"))+
theme(axis.text= element_text(face = "bold", color = "black", size = 11))+
theme(legend.title = element_text(colour="black", size=12, face="bold"),
legend.text = element_text(colour="grey0", size=11, face="bold"))+
theme(strip.text.x = element_text(size = 12,face="bold",colour = "black"))+
theme(strip.background = element_rect(fill = "lightblue", color = "black", size=1.5))
```

# Genome D

```
BIN_D<-read.csv(file="BINS_GROUPD.csv", header=T)
str(BIN_D)
BIN_D<-ggplot(BIN_D,aes(x=Position))+geom_bar(fill="brown2",color="brown2",
width=.3)+
theme_bw()+
facet_grid(.~Chromosome,scales = "fixed", space = "fixed") +
geom_rug(sides="b", col="black", size=0.6)+
```

```
labs(title = "", x = "Position of bin (cM)", y = "Markers per bin")+
theme (plot.title = element_text(color="black", size=14, face="bold"),
axis.title.x = element_text(color="black", size=16, face="bold"),
axis.title.y = element_text(color="black", size=16, face="bold"))+
theme(axis.text = element_text(colour = "black"))+
 theme(axis.text= element_text(face = "bold", color = "black", size = 11))+
theme(legend.title = element_text(colour="black", size=12, face="bold"),
legend.text = element_text(colour="grey0", size=11, face="bold"))+
theme(strip.text.x = element_text(size = 12,face="bold",colour = "black"))+
theme(strip.background = element_rect(fill = "lightblue", color = "black", size=1.5))
#Combine and plot all the three
 library(ggplot2)
 library(grid)
 library(dplyr)
 require(gridExtra)
# Save file in working folder
 tiff(file="BIN_ALL.tiff", width=10, height = 10, units = 'in',res=300,compression="lzw")
 BIN_ALL<-grid.arrange(BIN_A,BIN_B,BIN_D)
 dev.off()
```

R scripts used to plot distribution of segregation distortion markers and regions (Fig.3)

```
rm(list=ls())
setwd("C:/Users/wasim/Box Sync/DataAnalysis_ALL/GenotypicDataanalysis/LinkageMapping/Rqtl/Segregation_distortion")
map<-read.csv(file="SD_distribution.csv")
str(map)
# Draw the map

map$chromosome<-as.factor(map$chromosome)
#par(bg="white")
nb_K=nlevels(map$chromosome)
num=0
tiff("SD_ALL.tiff", width = 6.5, height = 6.5, units = 'in', res = 300, compression="lzw")
par(mar=c(4,4,1.2,1.2))
par(mgp=c(1.8, 0.5, 0))
plot(0,0,xlim=c(1,nb_K),yaxt="n",ylim=rev(range(map$position)),col.lab="black",ylab=
"Genetic distance (cM)", font.lab=2, cex.lab=1.5, bty="n",xaxt="n" , yaxt="n")
abline(h=seq(10 , max(map$position) ,10) , col="white", lwd=2)
for (i in levels(map$chromosome)){
 num=num+1
#text(num -0.01 , -0.8, i)
a=map[map$chromosome==i , ]
 points( rep(num,nrow(a)) , a$position , pch="-" , cex=1.9, col=ifelse
(a$type=="SD","forestgreen", ifelse(a$type=="SD30","red", "blueviolet")))}
par(font=2)
```

```
# add legend
legend("bottomright", legend = c("Normal SNP markers" , "SDRs (3-30 markers)", "SDRs
(>30 markers)" ) , col = c("blueviolet","forestgreen", "red"),
pch="-" , pt.cex =3, cex = 1.2, horiz = FALSE, bty="n", inset = c(0.07, 0.0001))
axis(2, at = NULL, labels = TRUE, tick = TRUE,font=2, col.axis = "black")
dev.off()
```

R scripts used to plot the marker order relationships between HW GBS linkage map and POPSEQ-based linkage map (Fig.4)

```
# Set working directory and read the data file
rm(list=ls())
setwd("C:/Users/whussain2/BoxSync/DataAnalysis_ALL/GenotypicDataanalysis/Linkag
eMapping/Linkagemap comparision")
data<-read.csv(file="Map_com.csv", header=T)
str(data)
library(ggplot2)
#Draw the plot
ggplot(data, aes(PosHW, PosPOP),aes(colour = factor(cyl))) + geom_point(size=2,
colour="purple")+
facet_wrap(~chrM,ncol=6)+theme_bw() +
theme(axis.text.x = element_text(colour = 'black', face="bold", size = 12, vjust=0.5)) +
theme(axis.text.y = element_text(colour = 'black', face="bold", size = 12)) +
 theme(axis.title.x = element_text(colour = 'black', face="bold", size = 22, vjust=-0.25)) +
theme(axis.title.y = element_text(colour = 'black', face="bold", size = 22, angle=90,
vjust=1.5))+xlab("HW GBS map (cM)") +ylab("POPSEQ map (cM)") +
theme(strip.text.x = element_text(size = 15, face="bold", colour = "black"))+
theme(strip.background = element_rect(fill = "lightblue", color = "black", size=1.5))
#Save the above plot
ggsave("R_HW_vs_POPSEQ.tiff", dpi=300, width=12, height=8.5, units="in",
compression="lzw")
```

R scripts used to plot SD marker proportions (Fig. 5)

```
setwd("C:/Users/wasim/BoxSync/DataAnalysis_ALL/GenotypicDataanalysis/LinkageM
apping/Rqtl/SD_proportions")
rm(list=ls())
SD<-read.csv(file="SD_markerprop_groupA.csv", header=T)
str(SD)
library(ggplot2)
#GenomeA
SD_prop_GenomeA<-ggplot(SD, aes(Marker, prop, group=Parents, color=Parents)) +
geom_line(lwd =0.8)+
facet_grid(.~Chromosome,scales = "free", space = "fixed")+
theme_bw()+
geom_rug(sides="b", col="black", size=0.6)+
```

```
labs(title = "", x = "Genetic position (cM)", y = "Proportion of allele")+
theme (plot.title = element_text(color="black", size=14, face="bold"),
axis.title.x = element_text(color="black", size=16, face="bold"),
axis.title.y = element_text(color="black", size=16, face="bold"))+
theme(axis.text = element_text(colour = "black"))+
theme(axis.text= element_text(face = "bold", color = "darkblue", size = 11))+
theme(legend.title = element_text(colour="black", size=12, face="bold"),
legend.text = element_text(colour="grey0", size=11, face="bold"))+
theme(strip.text.x = element_text(size = 14,face="bold",colour = "black"))+
theme(strip.background = element_rect(fill = "lightblue", color = "black", size=1.3))
```

#Genome B

```
SD<-read.csv(file=" SD_markerprop_groupB.csv", header=T)
str(SD)
library(ggplot2)
SD_prop_GenomeB<-ggplot(SD, aes(Marker, prop, group=Parents, color=Parents)) +
geom_line(lwd =0.8)+
facet_grid(.~Chromosome,scales = "free", space = "fixed")+
 theme_bw()+
 geom_rug(sides="b", col="black", size=0.6)+
 labs(title = "", x = " Genetic position (cM)", y = "Proportion of allele")+
 theme (plot.title = element_text(color="black", size=14, face="bold"),
 axis.title.x = element_text(color="black", size=16, face="bold"),
 axis.title.y = element_text(color="black", size=16, face="bold"))+
 theme(axis.text = element_text(colour = "black"))+
 theme(axis.text= element_text(face = "bold", color = "darkblue", size = 11))+
 theme(legend.title = element_text(colour="black", size=12, face="bold"),
 legend.text = element_text(colour="grey0", size=11, face="bold"))+
 theme(strip.text.x = element_text(size = 14,face="bold",colour = "black"))+
 theme(strip.background = element_rect(fill = "lightblue", color = "black", size=1.3))
```

# Genome D

```
SD<-read.csv(file=" SD_markerprop_groupD.csv", header=T)
str(SD)
library(ggplot2)
SD_prop_GenomeD<-ggplot(SD, aes(Marker, prop, group=Parents, color=Parents)) +
geom_line(lwd =0.8)+
facet_grid(.~Chromosome,scales = "free", space = "fixed")+
theme_bw()+
geom_rug(sides="b", col="black", size=0.6)+
 labs(title = "", x = " Genetic position (cM)", y = "Proportion of allele")+
 theme (plot.title = element_text(color="black", size=14, face="bold"),
 axis.title.x = element_text(color="black", size=16, face="bold"),
 axis.title.y = element_text(color="black", size=16, face="bold"))+
 theme(axis.text = element_text(colour = "black"))+
 theme(axis.text= element_text(face = "bold", color = "black", size = 11))+
 theme(legend.title = element_text(colour="black", size=12, face="bold"),
 legend.text = element_text(colour="grey0", size=11, face="bold"))+
```

```
    theme(strip.text.x = element_text(size = 14,face="bold",colour = "black"))+
    theme(strip.background = element_rect(fill = "lightblue", color = "black", size=1.3))
# Combine all plots
    library(ggplot2)
    library(grid)
    library(dplyr)
    require(gridExtra)
    tiff(file ="SD_proportions.tiff", width =12,height=10,units='in',res=300,
    compression="lzw")
    SD_proportions_ALL<-
    grid.arrange(SD_prop_GenomeA,SD_prop_GenomeB,SD_prop_GenomeD)
    dev.off()
```

R scripts used to plot LOD profile for CC, CW, FLRTs (Figure 6 and 7)

For CC and CW

```
# set working directory and read the data file
    rm(list=ls())
    setwd("C:/Users/waseem/Desktop/Plot/wax_chaff")
    CW<-read.csv(file="chaff_wax.csv")
    str(CW)
    chaff<-subset(CW, Trait=="Chaff color(CC)")
    str(chaff)
    CW$Position<-as.numeric(CW$Position)
    library(ggplot2)
    p<-ggplot(CW, aes(x = Position, y = LOD, col=Trait)) +
    geom_line(lwd =0.8)+
    theme_bw()+
    geom_hline(yintercept = 3, color = "darkred", size =1,show.legend = TRUE,linetype =2)+
# add thrshold hold for significance
    labs(title = "", x = "Genetic distance (cM)", y = "LOD score")+
    theme (plot.title = element_text(color="black", size=16, face="bold"),
    axis.title.x = element_text(color="black", size=14, face="bold"),
    axis.title.y = element_text(color="black", size=14, face="bold")) +
    theme(axis.text = element_text(colour = "black"))+
    theme(axis.text= element_text(face = "bold", color = "black", size = 12))+
    theme(legend.title = element_text(colour="black", size=10, face="bold"),legend.position
    = c(0.18, 0.85), legend.text = element_text(colour="grey0", size=10, face="bold"))
    p1<-p+facet_grid(.~ Chromosome,scales = "free", space = "free")+geom_rug(sides="b",
    col="black", size=0.6)+
    theme(strip.text.x = element_text(size = 12,face="bold",colour = "black"))+
    theme(strip.background = element_rect(fill = "lightblue", color = "black", size=1.5))
    #Add text to plot
    LodProfile1<-p1+annotate("text", x = 30, y=21, hjust=0.78, fontface="bold",
    colour="black", size=3, label = c("PVE(%) = 62.80", "PVE(%) = 48.90"))+
```

```
annotate("text", x = 30, y=19.5, hjust=1.2, fontface="bold", colour="black", size=3,label = c("AE = 1.06", "AE  = 0.26"))+
annotate("text", x = 28.8, y=18.1, hjust=0.6,fontface="bold", colour="black", size=3, label=c("CI=111.5cM-112cM","CI=4.5cM-5.50cM"))
ggsave(file="Lodprofile_cc_cw.tiff", width =8, height =4, units = "in", dpi=300,compression="lzw")
```

For FLRTs

# FLL

```
rm(list=ls())
setwd("C:/Users/waseem/Desktop/Plot/FlagL_qtl")
FlagL<-read.csv(file="FlagL_QTL.csv")
str(FlagL)
FlagL$Environment<-as.factor(FlagL$Environment)
FlagL$Position<-as.numeric(FlagL$Position)
library(ggplot2)
library(grid)
pl<-ggplot(FlagL, aes(x = Position, y = LOD.score, col = Environment)) +
geom_line(lwd =0.8)+
 theme_bw()+
geom_hline(yintercept = 2.8, color = "darkred", size =1.2,show.legend = TRUE,linetype = 2)+
labs(title = "(a)", x = "Genetic distance (cM)", y = "LOD score")+
theme (plot.title = element_text(color="black", size=20, hjust=0),axis.title.x = element_text(color="black", size=20, face="bold"),axis.title.y = element_text(color="black", size=20, face="bold")) +
theme(axis.text = element_text(colour = "black"))+
theme(axis.text= element_text(face = "bold", color = "black", size = 13))+
theme(legend.title = element_text(colour="black", size=14, face="bold"),legend.position = c(0.94, 0.78), legend.text = element_text(colour="black", size=11, face="bold"))
Lodprofile_FL<-pl+facet_grid(.~ chr,scales = "free", space = "free")+geom_rug(sides="b", col="black", size=0.3)+
theme(strip.text.x = element_text(size = 16.5,face="bold",colour = "black"))+
theme(strip.background = element_rect(fill = "lightblue", color = "black", size=1.5))
```

#FLW

```
#rm(list=ls())
FB<-read.csv(file="FlagB_QTL.csv")
str(FB)
FB$Position<-as.numeric(FB$Position)
str(FB)
library(ggplot2)
pb<-ggplot(FB, aes(x = Position, y = LOD.score, col = Environment)) + guides(colour=FALSE)+
geom_line(lwd =0.8)+
theme_bw()+
```

```
geom_hline(yintercept = 2.8, color = "darkred", size =1.2,show.legend = TRUE,linetype
= 2)+
labs(title = "(b)", x = "Genetic distance (cM)", y = "LOD score")+
theme (plot.title = element_text(color="black", size=20, hjust=-0.02),axis.title.x =
element_text(color="black", size=20, face="bold"),axis.title.y =
element_text(color="black", size=20, face="bold")) +
 theme(axis.text = element_text(colour = "black"))+
 theme(axis.text= element_text(face = "bold", color = "black", size = 13))+
theme(legend.title = element_text(colour="black", size=14, face="bold"),legend.position
= c(0.92, 0.8), legend.text = element_text(colour="black", size=12, face="bold" ))
Lodprofile_FB<-pb+facet_grid(.~ chr,scales = "free", space = "free")+
geom_rug(sides="b", col="black", size=0.4)+
theme(strip.text.x = element_text(size = 16.5,face="bold",colour = "black"))+
theme(strip.background = element_rect(fill = "lightblue", color = "black", size=1.5))
#For FLA
#rm(list=ls())
FA<-read.csv(file="FlagA_QTL.csv")
str(FA)
FA$Position<-as.numeric(FA$Position)
str(FA)
library(ggplot2)
# remove legend
pa<-ggplot(FA,aes(x=Position,y=LOD,col= Environment))+ guides(colour=FALSE)+
geom_line(lwd =0.8)+ # Draw as a line type
theme_bw()+ #use white theme
geom_hline(yintercept = 2.8, color = "darkred", size =1.2,show.legend = TRUE,linetype
= 2)+
labs(title = "(c)", x = "Genetic distance (cM)", y = "LOD score")+
theme (plot.title = element_text(color="black", size=20, hjust=0),axis.title.x =
element_text(color="black", size=20, face="bold"),axis.title.y =
element_text(color="black", size=20, face="bold")) +
theme(axis.text = element_text(colour = "black"))+
(axis.text= element_text(face = "bold", color = "black", size = 13))+
 theme(legend.title = element_text(colour="black", size=14, face="bold"),legend.position
= c(0.96, 0.93), legend.text = element_text(colour="black", size=12, face="bold" ))
Lodprofile_FA<-pa+facet_grid(.~ Chromosome,scales = "free", space =
"free")+geom_rug(sides="b", col="black", size=0.1)+
theme(strip.text.x = element_text(size = 16.5,face="bold",colour = "black"))+
theme(strip.background = element_rect(fill = "lightblue", color = "black", size=1.5))
#Combine all the plots
library(ggplot2)
library(grid)
library(dplyr)
require(gridExtra)
tiff(file = "LODprofile_ALLFLAG.tiff", width = 12.5, height = 12.5, units = 'in',res=300,
compression = “lzw”)
```

```
LODprofile_ALLFLAG<-grid.arrange(Lodprofile_FL,Lodprofile_FB,
Lodprofile_FA)dev.off()
```

R scripts used to plot distribution of markers on wheat genomes (Supplementary Fig. 4)

```
rm(list=ls())
setwd("C:/Users/wasim/Box
Sync/DataAnalysis_ALL/GenotypicDataanalysis/LinkageMapping/Rqtl/stackbar_plots")
library(RColorBrewer)
library(ggplot2)
genomes<-read.csv(file="Genome_all.csv")
str(genomes)
#plot the stacked bar plot with polar coordinates
tiff("genome.tiff", height=8, width=8, units = "in", res=300)
 ggplot(genomes, aes(x = Features, fill="variable")) +
 theme_bw()+
 theme( axis.title.x=element_blank())+
 guides(fill = guide_legend(title = "Genomes"))+
 geom_bar(aes(weight=numbers,fill=Genomes),position='fill')+
scale_y_continuous("",breaks=NULL)+ coord_polar()+
theme(axis.text.x=element_text(colour="darkred",size=12,angle=0,hjust=50,vjust=0,face
="bold"))+
theme(legend.title = element_text(colour="darkred",size=14, face="bold"), legend.text =
element_text(colour="Red", size=12, face="bold" ))
  dev.off()
```

Note: More details about the R scripts used to plot all the graphs above can be found in ggplot2 package (https://cran.r-project.org/web/packages/ggplot2/ggplot2.pdf), ggplot2 book (http://link.springer.com/book/10.1007%2F978-0-387-98141-3), and book "Guide to create beautiful graphics in R" (http://www.sthda.com/english/download/3-ebooks/5-guide-to-create-beautiful-graphics-in-r-book-get-a-free-pdf-copy/).

Supplementary References

1. Broman KW (2014) qtl: Tools for Analyzing QTL Experiments. R package version 135-3.
2. Taylor J, Butler D. (2016). ASMap: Linkage map construction using the MSTMap algorithm. R package version 0.4-7. 2016. Available: https://cran.r-project.org/web/packages/ASMap/index.html
3. Meng, L., Li, H., Zhang, L. & Wang, J. QTL IciMapping: Integrated software for genetic linkage map construction and quantitative trait locus mapping in biparental populations. *Crop J.* **3**, 269–283 (2015).
